# PLASTAMINATION: Outcomes on the Central Nervous System and Reproduction

**DOI:** 10.2174/1570159X22666240216085947

**Published:** 2024-02-16

**Authors:** Antonietta Santoro, Marianna Marino, Laura N. Vandenberg, Marta Anna Szychlinska, Erwin Pavel Lamparelli, Federica Scalia, Natalia Della Rocca, Raffaella D’Auria, Grazia Maria Giovanna Pastorino, Giovanna Della Porta, Francesca Felicia Operto, Andrea Viggiano, Francesco Cappello, Rosaria Meccariello

**Affiliations:** 1 Department of Medicine, Surgery and Dentistry “Scuola Medica Salernitana”, University of Salerno, 84081 Baronissi, SA, Italy;; 2 Department of Environmental Health Sciences, School of Public Health & Health Sciences, University of Massachusetts Amherst, Amherst, MA, USA;; 3 Faculty of Medicine and Surgery, Kore University of Enna, Cittadella Universitaria 94100 Enna (EN), Italy;; 4 Euro-Mediterranean Institute of Science and Technology (IEMEST), 90139 Palermo, Italy;; 5 Child and Adolescence Neuropsychiatry Unit, Department of Medicine, Surgery and Dentistry, University of 84100 Salerno, Salerno, Italy;; 6 Department of Science of Health School of Medicine, University Magna Graecia 88100 Catanzaro, Italy;; 7 Department of Biomedicine, Neuroscience and Advanced Diagnostics, University of Palermo, Palermo, 90127, Italy;; 8 Department of Movement and Wellness Sciences, Parthenope University of Naples, 80133 Naples, Italy

**Keywords:** Microplastics, nano plastics, brain, neuroinflammation, neurotoxicity, gonads, gametes, reproduction

## Abstract

**Background:**

Environmental exposures to non-biodegradable and biodegradable plastics are unavoidable. Microplastics (MPs) and nanoplastics (NPs) from the manufacturing of plastics (primary sources) and the degradation of plastic waste (secondary sources) can enter the food chain directly or indirectly and, passing biological barriers, could target both the brain and the gonads. Hence, the worldwide diffusion of environmental plastic contamination (PLASTAMINATION) in daily life may represent a possible and potentially serious risk to human health.

**Objective:**

This review provides an overview of the effects of non-biodegradable and the more recently introduced biodegradable MPs and NPs on the brain and brain-dependent reproductive functions, summarizing the molecular mechanisms and outcomes on nervous and reproductive organs. Data from *in vitro*, *ex vivo*, non-mammalian and mammalian animal models and epidemiological studies have been reviewed and discussed.

**Results:**

MPs and NPs from non-biodegradable plastics affect organs, tissues and cells from sensitive systems such as the brain and reproductive organs. Both MPs and NPs induce oxidative stress, chronic inflammation, energy metabolism disorders, mitochondrial dysfunction and cytotoxicity, which in turn are responsible for neuroinflammation, dysregulation of synaptic functions, metabolic dysbiosis, poor gamete quality, and neuronal and reproductive toxicity. In spite of this mechanistic knowledge gained from studies of non-biodegradable plastics, relatively little is known about the adverse effects or molecular mechanisms of MPs and NPs from biodegradable plastics.

**Conclusion:**

The neurological and reproductive health risks of MPs/NPs exposure warrant serious consideration, and further studies on biodegradable plastics are recommended.

## 1 INTRODUCTION

Plastic contamination (PLASTAMINATION) currently represents the main anthropogenic change in the global biosphere. Plastics are industrially made with the inclusion of dyes, pigments and different kinds of additives to get specific characteristics like strength, softness, or flexibility. They are largely used for the production of daily-use goods, including food and drink containers, single-use goods, clothes, personal care products, *etc*. Consequently, plastic wastes are diffused in the aquatic and terrestrial environment, but also in air samples. Depending on environmental conditions, plastic wastes are fragmented into small pieces (secondary source).

Commonly named microplastics (MPs) in the size range 1-1000 µm and nanoplastics (NPs) in the size range 1-1000 nm [1]. MPs and NPs have different shapes (mainly fibres or spheres), and a heterogeneous composition may represent a vehicle or a sponge for pollutants and or other chemicals capable of interfering in the endocrine signaling system (*e.g*., endocrine disrupting chemicals) responsible for the control of tissue homeostasis and biological functions [1-9]. In addition, due to their hydrophobic surface that encourages microbial colonization and biofilm formation, MPs serve as pelagic habitats for microorganisms and as vectors for the long-distance transmission of pathogenic bacteria [10]. Originally, PLASTAMINATION was considered an environmental trouble only. Hence, studies carried out in aquatic organisms revealed toxicity following plastic ingestion, but also developmental, neurotoxic or reproductive effects [3, 11-14]. In parallel, *in vitro* and *in vivo* studies revealed the ability of MPs/NPs to be internalized in cells and that directly or indirectly MPs/NPs enter the food chain and bypass the biological barriers, causing systemic exposure and bio-accumulation in liver, kidney, gonads or brain [15-18]. Hence, the last 5-years expansion of studies on terrestrial animals and the still unravelled queries make PLASTAMINATION both a diffuse environmental trouble and a possible health risk. Recently, the detection and the accumulation of MPs have been demonstrated in the tissues of terrestrial organisms, including humans, such as the brain, blood, placenta, gonads and semen [15, 18, 19]. In this review article, we focus on the outcomes in the central nervous system (CNS) and reproduction, first providing a general classification of plastics, plastic sources, exposure routes to target the brain and toxicant disposition. Ingestion is the main exposure route to MPs in vertebrates, and the preservation of the microbiota-gut-brain axis is fundamental to prevent psychiatric, neurodevelopmental, age-related, and neurodegenerative disorders [20, 21]. Hence, by means of a comparative approach, we report the effects of non-biodegradable and the more recently introduced biodegradable MPs and NPs in the brain on non-mammalian (*i.e*., zebrafish, both the forefront of toxicology research and a prominent vertebrate model for disease) and mammalian vertebrates. Pubmed search from 2012 to 2023 with the keywords micro(nano)plastics in combination with the brain (387 items), reproduction (192 items), zebrafish (30 items) and mammals (16 items) were used. From the analyzed literature, the main consequences of MPs/NPs exposure in the brain are neurotoxicity, de-regulation of synaptic-functions, microglia activation and neuroinflammation followed by alterations of blood-brain barrier (BBB) permeability, situations that, if long-time prolonged is at the basis of age-related neurological diseases like the Alzheimer disease and the Parkinson disease [22-24]. Lastly, we discuss the effects of MP exposure on brain-dependent reproductive functions, revealing the imbalance of the hypothalamus-pituitary-gonadal axis [9], poor gamete quality and successful reproduction as endpoints. Data from *in vitro*, *ex vivo*, non-mammalian and mammalian animal models, but also epidemiological studies have been reviewed and discussed pointing out that the neurological and reproductive health risks of MPs/NPs exposure warrant consideration and further studies on the health safety of biodegradable plastics are recommended.

## 2 CLASSIFICATION OF PLASTICS, SOURCE, EXPOSURE ROUTES AND TOXICANT DISPOSITION

### 2.1 Classification, Chemical Characteristics and Industrial Applications of Plastic Materials

The term “Plastic” means something that can be shaped into various forms [25], giving a double meaning to consumer plastics because the development of plastic materials and plastic polymers has continued and evolved over decades. In fact, due to their remarkable versatility and usefulness, plastics have become a vital material in almost every aspect of human life. All plastics are substantially grouped into two categories: fossil fuels, which are often referred to as conventional plastics, and the more recently introduced bioplastics that are manufactured with renewable sources (not conventional) [26]. Both kinds of plastic can be either biodegradable or not biodegradable. Plastics that are not biodegradable consist of backbone polymers that are resistant to hydrolysis and biodegradation [27], whereas microorganisms can easily break down biodegradable plastic. The types of the most well-known plastics are given in Fig. (**1**).

Among conventional plastics, Polyolefins can derive from petrochemical or renewable resources (*e.g*., bioethanol from sugar cane) being both biodegradable or non-biodegradable [28]. They are thermoplastic polymers, in which olefin monomer units like ethylene, styrene, and vinyl chloride are combined to form long chains of polymers [29, 30]. Polyolefins such as Polyethylene (PE) and Polypropylene (PP) represent the leading and largest industrial volume polymers since they are responsible for over 50% of the world's plastic production and more than 90% of packaging materials due to their exceptional chemical stability and mechanical properties [31]. However, many other types of plastic materials are produced for a variety of uses, such as Polyvinyl chloride (PVC) and Polystyrene (PS). Due to contamination of the environment with these plastics, several other biodegradable and “environmentally sustainable” plastics have been developed. Chemical characteristics and the most widespread applications of plastics on the market are summarized in Table **1** [32-75].

Conventional as well as bio-based and biodegradable plastics can contain several chemical compounds that are used to improve their functional properties. The most common additives, also known as intentionally added substances (IAS) [76], include plasticizers (phthalates), flame retardants, antioxidants (like IRGAFOS-168), acid scavengers, mechanical stabilizers (like bisphenols), pigments, antistatic agents, slip compounds, and thermal stabilizers [77]. These IAS can degrade and generate non-intentionally added substances (NIAS). Indeed, NIAS could also be occasionally generated from contaminants present in the equipment or during the manufacturing process, as in the case of oligomers or monomers formed from polymeric feedstock (*e.g*., PS oligomers) [78, 79]. However, their presence, identity and amount are not known by the producer nor by the consumer. Because NIAS, as well as many IAS, are not covalently bound to the polymer, they can be released [79, 80] and migrate into food, articles, liquids or the environment, posing a risk to human health and potentially contributing to environmental pollution [77, 81-83]. In addition, while the potential toxicity of some of these substances is known, as is the case for bisphenols like BPA and phthalates [84-86], for most NIAS, the health risks that might derive from their exposure are not known, and it is often challenging to identify these chemicals in the first place [79]. For this reason, further investigations are needed to replace these additives with safer, more sustainable and environmentally friendly alternatives [77]. With increased consumer demand for environmentally friendly solutions, bioplastics are considered a valid alternative, but their development has several limitations, such as high energy and cost expenditures for their production, lower mechanical properties and degradation rate depending on specific environmental conditions, and relatively poorly studied toxicity for the chemicals used in their production [26, 87, 88].

### 2.2 Contamination Routes and Accumulation in the Brain

Growing evidence has highlighted the inauspicious effect of MPs/NPs on a wide range of organisms, including plants, fish, plankton, microorganisms and rodents [89]. However, the ubiquitous presence of plastics in everyday consumer products (detailed in Table **1**) implies the inevitable exposure of humans to MPs. In fact, studies have revealed the presence of MPs in human lungs, stool and placenta [18, 90]. The potential hazards and health implications of MPs/NPs for humans are, therefore, a matter of concern. The possibility that particles can enter living organisms internalize in cells and/or migrate far away from the primary exposed tissues to target secondary tissues depends on their size but also on their shape and chemical properties, such as hydrophobicity and surface charge. There is evidence that MPs < 5 µm can enter the main circulation from the gut and accumulate in the brain, liver, and kidney; MPs/NPs that are 0.1-10 µm cross biological barriers like BBB and placenta and bio-accumulate in secondary tissues including brain and liver [9].

Inhalation represents the main human exposure route for MPs/NPs released from plastics, textiles, and synthetic tire wear in airborne [91, 92]. These particles, spread through the air, could induce lung inflammation and fibrosis [93]. Particularly due to tire abrasion on roadways, PS-MPs, which are the most relevant plastic polymer found in tire wear [93], are released into the environment. It has been shown that tire wear-derived PS-MPs can increase pro-inflammatory cytokines such as interleukin (IL)-6, IL-8, tumor necrosis factor- α (TNF-α) [94], and induce oxidative stress, genotoxicity, pulmonary fibrotic injury, and diminished ventilatory function [95, 96]. However, toxicological data on the inhalator effects of tire wear are currently few. Hence more extensive research should be conducted to deeply investigate the impact of tire wear-derived MPs on human health.

The inhalation and nasal route have been used for drug delivery to the brain [97], leading to speculation that when MPs/NPs are unintentionally inhaled, the olfactory nerve pathway provides an entry portal to the central nervous system. Various nanomaterials, once in contact with the olfactory epithelium, can be transported to the brain through olfactory neurons to induce detrimental effects like brain inflammation [98-100]. However, few studies have investigated this route of exposure, even after confirmation of the presence of MPs and NPs in the respiratory system and their passage across the BBB [92, 101].

Ingestion is another significant exposure route, as confirmed by the presence of MPs in drinking water, drink containers and foods, including crustaceans, molluscs and fish [102-104] and by their elimination in stool [90]. After ingestion, PS particles accumulate in mouse organs, including the intestine, liver, kidney, testes and brain [105]. Quantitative detection of PS distribution *in vivo*, using fluorescence colorimetry, revealed a higher accumulation of PS MPs in the brain. Accumulation in tissues was confirmed using a small animal imaging system to measure the fluorescence intensity of tissue [106]. The potential pathways by which PS-MPs can enter the brain by ingestion include translocation through the BBB (which may have enhanced permeability following PS-MP treatment) and lymphatic or systemic circulation in brain regions lacking the BBB [107, 108]. However, studies examining the mechanisms by which MPs and NPs enter the brain remain preliminary and are limited to only a few kinds of plastics, hence the need for further investigation.

Fig. (**2**) summarizes plastic sources, types, diffusion, exposure routes and targets for living organisms.

## 3 PLASTICS-INDUCED EFFECTS ON CNS

### 3.1 Neuroinflammation as a Hallmark of Neurotoxicity: An Overview

Neuroinflammation is a multifaceted process involving the activation of glial cells, mainly astrocytes and microglia, and the release of pro-inflammatory mediators in response to injury or infection. The inflammatory response is protective for brain tissue; however, an exacerbated or prolonged inflammatory response by astrocytes can be detrimental, leading to tissue damage, neuronal loss and, finally, neurodegeneration [109, 110]. In this respect, astrogliosis is the transcriptional reprogramming of astrocytes that triggers cellular hypertrophy, proliferation, production of inflammatory mediators and secretion of reactive oxygen species (ROS) through the activation of different pathways, including NFkB [24].

Beyond their direct role in CNS immune response, astrocytes possess two other important functions: i) astrocytes endfeet line the basement membrane of blood vessels endothelial cells forming the BBB, which delimits the perivascular space [111]; ii) astrocytes control the entering of substances and pathogens to the brain parenchyma and regulate water homeostasis and the trafficking of proteins involved in bidirectional fluid exchanges. Therefore, under inflammatory conditions, morphological and metabolic changes in astrocytes [112, 113] can alter the homeostasis of this complex microenvironment, impairing BBB integrity and allowing peripheral immune cells and plasma proteins to extravasate [112, 113]. Accordingly, infiltrated immune cells can establish cell-to-cell contacts with astrocytes to activate the transmigration of peripheral immune cells and promote neuroinflammation [114]. Alterations of BBB permeability with activation of astrocytes and microglia have been observed in Traumatic Brain Injury (TBI), Multiple Sclerosis (MS), Amyotrophic Lateral Sclerosis (ALS) and Alzheimer’s disease [113, 115-117]. Furthermore, a number of studies in experimental animal models of neuroinflammation and neurodegeneration showed increased levels of monomeric amyloid‐β (Aβ) in brain homogenates [118], BBB alterations, decreased expression of synaptic markers and higher expression of pro‐inflammatory cytokines frequently associated with demyelination and neuronal death [119]. However, the precise molecular events leading from neuroinflammation to a specific neurological or neurodegenerative disease are unknown. Similarly, it is not clear whether BBB injury precedes or advances neuroinflammation. A key finding in understanding the dynamics of neuroinflammation has been the discovery of a glial-associated functional homologue of the lymphatic system in the brain named the g-lymphatic system [120, 121] that facilitates the movements of cerebrospinal fluid (CSF) and interstitial fluid (ISF) in and out of the brain [120]. Downstream of the g-lymphatic system is the meningeal lymphatic system, representing a new drainage path for CSF-contained macromolecules and immune cells to exit from the CNS and reach the cervical lymph nodes and the peripheral immune system [122, 123]. Various neurological disorders like TBI and Alzheimer’s disease show alterations of CSF flow; thus, it has been proposed that g-lymphatic impairment could augment neuroinflammation by suppressing cytokine clearance and removal of waste products from the brain [124, 125]; accordingly, the enhancement of meningeal lymphatic drainage can ameliorate neuroinflammation [126, 127].

In the progression or resolution of inflammation, intercellular cross-talk between astrocytes and microglia is a key regulator: several research groups have identified microglia-derived molecules such as IL-1β, IL-10, TNF-α, vascular endothelial growth factor (VEGF)-β, or transforming growth factor (TGF)-α, among others, that can switch the transcriptional signature of astrocytes during neuroinflammation [128, 129]. On the other hand, in *in vitro* co-cultures, astrocytes cooperate with neurons to promote a more ramified morphology and induce expression of microglial signature genes, suggestive of a more mature *in vivo*-like phenotype [130]. Indeed, under physiological conditions, microglia are tightly controlled by the local microenvironment, thus, inflammatory stimuli change this niche, leading to an activated state referred to as “priming” [131]. Priming makes the microglia more susceptible to a secondary inflammatory stimulus, which can generate an exaggerated inflammatory response [132]. This abnormal inflammatory status contributes to the release of additional pro-inflammatory cytokines in the local environment, compromising the role of microglia in monitoring neural activation and synapse pruning. Disruption of the microglia-neuron interplay may alter neuron excitability and behaviour promoting the development of neurological disorders [133].

The consideration of the brain as a site of immune privilege has changed in recent years. It is now better understood that there is a continuous crosstalk between brain immunity and the peripheral immune system. Similarly, it is now accepted that there is bidirectional communication between the gastrointestinal tract and CNS through a specific network of signalling pathways (the gut-brain axis) comprising the vagus nerve, the immune system and the metabolites and molecules produced by intestinal bacteria [134]. Indeed, alterations of gut microbiota (dysbiosis) can affect the interplay between the gut and brain, perturbing immune homeostasis, altering BBB permeability, and finally activating neuroinflammation. Intestinal microbes belonging to *Lactobacillus, Bifidobacteria, Enterococcus,* and *Streptococcus* species produce neurotransmitters such as acetylcholine, γ-aminobutyric acid (GABA), serotonin, and its precursor tryptophan [135, 136] which influence the myenteric plexus and then transported to the CNS *via* the systemic vasculature interfere with glial cell behaviour [137, 138].

In view of factors modulating neuroinflammation and predisposition to neurodevelopmental disorders (NDD), studies carried out on Maternal Immune Activation (MIA) *in vivo* models have revealed that parental exposure to immunostimulants (*i.e*., viral mimic polyinosinic: polycytidylic acid, poly(I: C) and bacterial mimic lipopolysaccharide, LPS) increases pro-inflammatory cytokines in the placenta and foetal brain. The cytokines IL-6 and IL-17 seem to mediate this phenomenon [139, 140] and factors inducing MIA to activate the Toll-like receptors (TLR) pathway that, in turn, stimulates additional cytokine and chemokine production in target cells [141, 142]. The imbalance in cytokine production in the foetal brain might be responsible for an abnormal microglial and astrocyte signature leading to increased susceptibility to NDD. Indeed, the effects of MIA, mediated by acute and chronic inflammation in pregnancy, are transduced to the foetus through inflammatory cell signalling pathways. Environmental perturbations such as those affecting the microbiome or prenatal immune activation, lead to alteration in the microglia transcriptome signature [143] and impairment of phagocytic capability in offspring [144]. In addition, results from the MIA model provide evidence for sex-specific vulnerabilities to different inflammatory stimuli, suggesting a key role of inflammatory signalling molecules and the innate immune system in directing brain masculinization. These results suggest that the male brain may be more susceptible to MIA [145, 146].

From the above observations, it appears that environmental factors, including MPs and NPs, that induce ROS production and trigger inflammation could cause irreversible effects directly on exposed subjects and indirectly on the progeny.

### 3.2 Neurotoxicity of MPs and NPs in Brain

#### 3.2.1 Evidence from Non-mammalian Models: Focus on Zebrafish Danio Rerio

The study of neuroinflammation and neurotoxicity in animal models following exposure to environmental pollutants is laborious and time-consuming; the use of non-mammalian models offers some advantages due to their fast reproduction, rapid development and less expensive maintenance. One of the main vertebrate animal models used for neurotoxicity studies is the zebrafish (*Danio rerio*). Indeed, this small and transparent fish presents 71.4% homology with human genes and 82% homology with human genes relevant to morbidity [147, 148]. The zebrafish brain exhibits morphological and functional overlapping zones to those of humans, such as the presence of the cerebellum, telencephalon, diencephalon, spinal cord, and enteric-autonomic nervous systems [149-151]. Glial cells of adult zebrafish express similar human genes and proteins, such as the brain lipid-binding protein (BLBP), glial fibrillary acidic protein (GFAP), and the calcium-binding protein S100 (the GFAP corresponding marker in rodents) [152]. In addition, transcriptome analysis between a zebrafish amyloid toxicity model and the datasets of two human adult brains and one foetal brain showed that approximately 95.4% of the human and zebrafish cells were co-clustered [153]. Those clusters included 15 neuronal clusters (45.4% of all cells) and nine astroglial clusters (18.1% of all cells) [153]. Adult zebrafish brain also express pro-inflammatory cytokines (*e.g*., IL-4) [154], the brain-derived neurotrophic factor BDNF [152], and respond to the injection of human Aβ42 activating immune response and pro-inflammatory gene expression, and neuronal death in a way resembling the deposition effects induced by Aβ42 peptide in humans [154]. Therefore, brain zebrafish features highlight the good potential of this alternative system for neuroinflammation and neurotoxicological studies [16].

The inappropriate disposal of plastics has determined the release of their debris in aquatic environments, triggering an ecological risk to the present and future generations. There are several papers reporting the effects of MPs/NPs in aquatic eco-systems such as zooplankton, a principal food source for many secondary aquatic consumers [155], microalgae, the primary food source of all aquatic food chains [156] and several aquatic organisms such as mussel, crabs, marine worms and fishes which represents a possible route whereby plastic debris could be transferred up the trophic levels [155, 157]. The toxicological effects of NPs and MPs, mainly those derived from PS, PET and PVC, have been extensively studied in aquatic organisms and are shown to be dependent on plastic particle size, surface physicochemical characteristics, concentration and exposure time. Most of these studies revealed developmental toxicity, reproductive toxicity, neurotoxicity, locomotor impairment, immunotoxicity, intestinal damage, metabolic disorders and microbiome composition alterations after exposures [13, 157]. Most of the studies conducted in zebrafish demonstrated that oxidative stress, hypomobility, inflammatory responses, alterations in cholinergic, GABA-ergic and dopaminergic systems, nervous system-related gene expression disruption and histopathological neuronal damage are the most common effects of MPs exposure. In zebrafish, NPs administered *via* food penetrate the BBB, causing alterations to the mass and morphology of the brain tissue, as well as alterations in acetylcholinesterase activity, neurotransmitter levels and oxidative stress, which may contribute to altered behavioral patterns and locomotor impairment [158]. The neurobehavioral effects of PS-NPs (diameter size of ~70 nm), along with other forms of accumulation site-dependent toxicity, have recently been investigated in adult zebrafish [159]. NPs accumulated in the gonads, intestine, liver and brain and induced alterations in locomotion activity, aggressiveness, shoal formation, predator avoidance behavior, and dysregulated circadian rhythm locomotion activity, especially at high concentrations. These effects were associated with alterations in neuronal biomarkers such as acetylcholinesterase (AchE) activity, as well as alterations in dopamine (DA), acetylcholine (ACh), serotonin (5-HT), melatonin (MT) and GABA levels, already one week after NPs exposure. The neurotoxic effects of MPs exposure have similarly been investigated [160]. UV-aged PS-MPs (size = 1 μm) reduced the average swimming speed of zebrafish larvae and altered neurotransmitter and neurotransmitter-related molecule concentrations. Moreover, increased DA, 5-HT, GABA and ACh concentrations were observed 120h after exposure, suggesting that the neurobehavioral impairments were caused by neurotransmitter imbalance, as confirmed by other studies [159, 161, 162]. Similarly, the neurotoxicity of UV-aged PS-MPs was assessed in a recent study in zebrafish larvae [163]. Reduced locomotor behavior was again observed and correlated with the altered expression of neurotransmission- and oxidative stress-related genes. A comparison of UV-aged MPs with virgin MPs in developing zebrafish confirmed that photodegradation could alter the physicochemical properties of MPs, rendering them more toxic.

Furthermore, Umamaheswari *et al.* [164] demonstrated that PS-MPs, induced histopathological lesions, including inflammation, degeneration, necrosis and hemorrhage in zebrafish brain and liver along with the upregulation of *gstp1, hsp70l*, and *ptgs2a* and downregulation of *cat, sod1, gpx1a*, and *ache* gene expression, suggesting PS-MPs can induce different toxic effects by altering the metabolic mechanism, histological architecture and gene regulation pattern through ROS induced oxidative stress. In particular, it has been suggested that increased ROS generation by PS-MPs damages mitochondrial membranes and reduces oxidative phosphorylation, impairing ATP synthesis and leading to cell death/necrosis. Furthermore, ROS induced protein carbonylation and lipid peroxidation (of cell membrane proteins and lipids) results in altered membrane permeability, loss of membrane potential, and Damage-Associated Molecular Patterns (DAMPs) release. ROS-induced DNA oxidation determines transcriptional changes and negatively affects the translation of specific proteins. This leads to the inhibition of the activity of specific proteins, altering the physiological and behavioral responses of zebrafish [132].

Furthermore, in a study by Teng *et al.* [165], the exposure to amino-modified (positive charge) PS-NPs induced stronger developmental toxicity (decreased spontaneous movement, heartbeat, hatching rate, and body length), neuronal cell apoptosis and greater neurobehavioral impairment as compared to carboxyl-modified (negative charge) PS-NP. In particular, positively charged PS-NP decreased levels of glycine, cysteine, glutathione, and glutamic acid, and the interaction with the neurotransmitter receptor N-methyl-D-aspartate receptor 2B (NMDA2B), suggesting neurodegeneration due to glutamatergic synapses disruption. On the contrary, the negatively charged PS-NP increased levels of spermine, spermidine, and the biosynthetic precursors of tyramine and showed interaction with the G-protein-coupled receptor 1 (GPR1), inducing motoneuron excitability. Moreover, GPR1 was shown to modulate differentiation and proliferation of neural stem cells, with implications in neurodegenerative disease onset. The toxicity of NPs and MPs has also been assessed in other aquatic species. Freshwater invertebrate *Daphnia magna* exposed to PS-NPs through the food chain developed brain tissue swelling and locomotor impairment [166]. A mixture of environmentally diffused MPs (polyacrylamide, polyacrylic acid and one biopolymer, zein) decreased levels of acetylcholinesterase (AchE) and excessive lipid peroxidation (LPO) levels in the brains of wild fishes (*Dicentrarchus labrax, Platichthys flesus, Mugil cephalus*) caught from a contaminated estuary in North Atlantic, indicating that MPs diffused in the environment effectively cause neurotoxicity [167]. Other studies have similarly reported alterations in AchE activity in the brains of red tilapia exposed to PS-MPs for short periods [168]. In a recent meta-analysis [169], MPs-induced neurotoxicity was assessed in aquatic animals exposed to environmentally realistic MP concentrations (≤ 1 mg/L, median = 0.100 mg/L). MP exposure was consistently shown to decrease AchE concentrations in the brain, supporting that AchE activity alteration represents a principal neurotoxicity biomarker implicated in biological neurotransmission. Furthermore, its alteration was reported to result in a variety of clinical symptoms including weakness, sweating, vomiting, diarrhea, tremor, and gait disturbance, as well as death from respiratory or heart failure [170].

The neurotoxicity and behavioral changes due to NPs/MPs exposure might also be correlated with disturbances to intestinal flora through the dysregulation of the gut microbiota-brain axis, which itself consists of immune, neuronal, microbial and hormonal pathways impacting organism development and health [171]. Significant alterations have been observed in the gut microbiome due to oxidative stress, inflammation and lipid metabolism induction after 21 days of exposure to MPs in zebrafish [172]. Within 7 days of exposure, larvae exposed to PE-MPs have disrupted microbiomes and lipid metabolism [173]. Furthermore, PS-NPs disrupt the brain-intestine-microbe axis after exposure during embryo-larval development in zebrafish [174]. Effects on inflammatory responses, intestinal permeability and growth inhibition were observed in larvae after 30 days of exposure, and targeted metabolomics analysis revealed an alteration in metabolites involved in neurotransmission, suggesting that PS-NPs are strongly linked to a disrupted regulation of the gut-brain axis, finally resulting in the impaired gut and brain functions.

Lately, bio-based plastic polymers are ideal alternatives to petroleum-based plastics because they can reduce the reliance on fossil fuel resources and many such polymers are degradable in the environment. Relatively few studies have evaluated the effects of MPs/NPs from bioplastics on aquatic organisms, although some recent studies in *Danio rerio* provide some evidence of neurotoxicity. For example, the effects of degradable MPs from PGA and PLA plastics were evaluated in zebrafish, and exposures to both bioplastics decreased survival and hatching rates, reduced voluntary locomotion, induced anxiety-like behaviors and impaired circadian rhythm in zebrafish larvae [175]. Moreover, some behavioral changes (in the shoal and anti-predatory defensive response deficit) and biochemical dysfunctions related to cholinergic system alterations have been demonstrated in adults as well as in larval zebrafish after PLA-MP exposure [176, 177]. In particular, the increased AchE activity, responsible for behavioral alterations (in shoal), and redox imbalance, featured by increased production of ROS, were documented by Chagas *et al.* [176]. Controversially, the study by de Oliveira *et al.* [177] reported that PLA bioMPs determined AchE activity inhibition, which was suggested to reinforce the accumulative potential of biopolymers and their direct or indirect role as anxiogenic agents, even at sublethal concentrations. Other studies have shown that PLA-MPs altered the diversity of intestinal microbiota, promoting bacterial species closely linked with energy metabolism, cellular processes, and fish diseases [178], providing a probable link with neurotoxic effects deriving from gut-brain axis crosstalk. Furthermore, like what has been observed with petroleum-based conventional plastics, photolytic degradation of PLA plastics elevates its toxicity in developing zebrafish, with effects triggered by mitochondrial dysfunction and apoptosis [179]. Importantly, the ecological risks derived from exposures to the middle- and end-products of the PLA biodegradation process, including their effects on organ development and function in aquatic organisms, still remain to be explored. A summary of the effects of MPs and NPs observed in zebrafish is summarized in Table **2** [180-201].

#### 3.2.2 Evidence from Mammalian Models

##### 3.2.2.1 *In vitro* Models

The neurotoxic effects of conventional, petroleum-based MPs and NPs have been explored in both cultured neurons and glia. Five murine neuronal cell types exposed to PS-NPs revealed that NPs can impact mitochondrial activity and lactate dehydrogenase (LDH) leakage in neuronal cells, although only at the highest concentration tested (250 mg/L) [202]. Furthermore, microglial cells could internalize carboxylated PS-NPs through phagocytosis, suggesting the potential for neuroinflammation, as observed following exposure to metal(oxide) nanoparticles. Interestingly, in contrast to carboxylated nanoparticles, microglial cells exhibited minimal internalization of PEGylated NPs [202], suggesting that the composition and physio-chemical properties of the plastics could impact the uptake of these particles into microglia.

Pro-inflammatory actions of MPs and NPs *via* the induction of cytotoxicity and inflammation in CNS-derived cells have also been demonstrated. Human T98G cerebral cells exhibited elevated production of ROS following 24h exposure to PS-MPs (10 μm, 0.05-10 mg/L), but only at the highest tested concentration (10 mg/L), whereas exposure to PET-MPs (3-16 μm, 0.05-10 mg/L) did not alter ROS production [203]. In a previous study, particle internalization was demonstrated with PET-NPs (size 33 nm) in human dopaminergic neurons and developing neurospheres [204] generated from early CNS PAX6(+) precursors and further differentiated within 3-D structures. PET NPs were tested in short exposure (48 h, 22.5-1440 mg/L) and chronic exposure (18 days, 22.5-360 mg/L): results showed that the internalization of PET-NPs coincided with altered gene expression and increased malondialdehyde (MDA) levels, indicative of oxidative stress. At high concentrations (≥180 mg/L), exposure decreased cell viability [204].

Two recent *in vitro* studies showed that PS-NPs could cross the BBB, inducing neuroinflammation in mammals by activating microglia cells. In the first, human cerebral microvascular endothelial cells were shown to internalize PS-NPs and trigger the generation of ROS, activation of NF-κB and secretion of TNF-α and disrupt tight junctions [205]. Exposure to PS-NPs also activated murine microglia BV2 cells, and the conditioned medium from PS-NPs-exposed BV2 cells caused significant damage to murine neuron HT-22 cells [205]. In the second study of the BBB, BV2 cells were shown to internalise PS-NPs, (12 and 24h, 25 to 100 mg/L) inducing inflammatory reactions (100 mg/L) and ferroptosis (50 and 100 mg/L) by increasing the Fe^2+^ concentration, also suggesting that the mechanism of action could occur *via* the c-Jun N-terminal kinase (JNK)/heme oxygenase (HO-1) pathway [206]. Activation of neuroinflammatory responses following MPs contamination has also been reported in human microglial HMC-3 cells treated with PS-NPs of different sizes (0.2, 2 and 10 μm) [207], with changes in cellular morphology, immune responses, and microglial apoptosis induced by PS-MPs phagocytosis. These effects may have been mediated by the activation of NF-κB-induced pro-inflammatory cytokines and associated induction of apoptotic markers. In addition, HMC-3 cell transcriptome analysis showed that PS-MPs treatment could change the expression of immune response genes, immunoglobulins, and several related microRNAs.

Similar outcomes were observed in human neuroblastoma cells (SH-SY5Y) following exposures to PS-NPs, which caused cytotoxicity, oxidative stress, LDH release and induced cell differentiation into a neuronal phenotype [208]. The observed effect was comparable to that of acrylamide, a well-recognized potent neurotoxin. Of note, PS-NP exposure induced shrinkage of neurite outgrowth, morphology alterations and swelling of the nuclei. Potential negative effects of PS-NPs on neurulation have also been investigated. In SH-SY5Y cells, PS-NPs were internalized *via* caveolae-mediated endocytosis [209]. Analysis of endocytic markers such as LC3B, Atg7, Atg5 and p62 revealed that autophagy was activated in SH-SY5Y cells exposed to PS-NPs. However, the cells were unable to degrade the PS-NPs, and the cytoplasmic accumulation of these particles inside SH-SY5Y cells caused faulty apoptotic cell death in the development of neural tubes [209]. Furthermore, SH-SY5Y cells treated with high concentrations (24h, 100 to 500 mg/L) of PS-NPs activated the mitochondrial apoptotic pathway in a concentration-dependent manner [210].

To study the effects of PS-MPs exposures on neural development, Hua *et al.* [211] utilized a 3D model of human forebrain cortical spheroids mimicking the early development of the human cerebral cortex. PS-NPs (from 1 to 10 µm, 5mg/L) accumulated during brain tissue embryonic development and adversely affected brain-like tissue development in a size- and concentration-dependent manner. In particular, a short (5-10d) exposure to PS-MPs enhanced cell proliferation and the expression of neural progenitor genes, while longer (5-30d) exposures decreased cell viability and downregulated neural differentiation markers. Interestingly, changes in the size and concentration of PS-MPs altered the expression of DNA damage and neural tissue patterning genes [211].

Unfortunately, few studies have evaluated the effects of bioplastics on cells derived from the CNS. In one, the neuroprotective effects of recombinant human erythropoietin (rhEPO)-loaded poly(lactic-co-glycolic acid) (PLGA) nanoparticles stabilized by sodium cholate (rhEPO-Ch-NP) were evaluated in SH-SY5Y cells [212]. No cytotoxic effects were observed; rather, the rhEPO-Ch-NP protected normocytic features from glutamate-induced neurotoxicity. Additional studies are needed to evaluate other bioplastic polymer MPs and NPs on the various cell types in the CNS. Furthermore, studying the effects of MPs and NPs on neurotoxicity is a complex task, and there are several challenges in this field that should be considered in the study of conventional plastics and bioplastics. These include the need for standardized methods to assess MPs and NPs in biological samples, understanding their mechanisms of interaction with the brain and immune system, and establishing relevant exposure scenarios that mimic real environmental conditions.

##### 3.2.2.2 *In vivo* Studies in Rodent Models

As reported above, few data are available on the neurotoxic effects of MPs and NPs in mammalian *in vitro* models, and most studies have focused on particles from PS plastics. Even fewer studies of either conventional and not non-conventional MPs/NPs have examined effects on mammalian CNS *in vivo*. Most of the *in vivo* studies have been carried out by using ingestion as the route of exposure and have examined the accumulation of MPs and neurotoxic effects on glia and neurons. The effect of MPs has been investigated in BALB/c mice administered PS-MPs in drinking water for 180 days [213]. Accumulation of PS-MPs in brain tissues was observed, as well as disruption of BBB integrity, reduction of spine density and inflammation. In the cornu ammonis 3 (CA3) and dentate gyrus (DG) regions of the brain, the mRNA levels of caspase 3 and the Bax/Bcl-2 mRNA ratios were significantly higher in exposed mice compared to controls, suggesting an increase in neuron apoptotic activity. Furthermore, a reduction in the spine density located in the hippocampal cornu ammonis 1 (CA1) region was observed together with lower expression of proteins involved in regulating synapse formation, such as synapsin 1, synaptophysin and PSD95. Notably, these effects are associated with the induction of a pro-inflammatory status, and the effects of PS-MPs were concentration-dependent but not particle size-dependent [213]. Lee and colleagues [214] also found that PS-MPs were able to cross the BBB after ingestion, with particles detected in the brain. RNA sequence analyses revealed that in the male hippocampus, the expression of several genes that play a role in regulating synaptic plasticity and neuronal activity were decreased. The effects found in the hippocampus were associated with alterations in learning and memory capacities and mediated through the vagus nerve-dependent pathway. Additionally, there were clear signs of neuroinflammation after 8 weeks of treatment, with an increase in the number and volume of microglia in several subregions of the hippocampus together with enhanced expression of microglial markers [214]. Similar results were obtained in mice fed PET-MPs (1.005g/cc, 10-20 μm) for 2 weeks [215]. In this case, gene expression variations were found in the prefrontal lobe and hippocampus regions. Changes in genes involved in endothelial cell chemotaxis, mitotic cell cycle arrest, and cell migration appeared in the prefrontal cortex, while in the hippocampus, the genes downregulated are involved in transcription, DNA synthesis, and angiogenesis. Notably, in both of these *in vivo* studies, oral ingestion of MPs caused significant alterations in the gut microbiome, especially in carbohydrate and lipid metabolism. This suggests that both PS- and PET-MPs exposure could induce adverse effects directly through accumulating in the CNS and indirectly by changing the gut-brain axis. Moreover, the observation that microbiome alterations were greater in animal models of Alzheimer's disease compared to healthy animals [215] provides some evidence for increased susceptibility to MPs in patients affected by neurological/neurodegenerative disorders compared to healthy subjects.

In another study, Wistar rats administered orally with low-density PET-MPs (2 µm, 0.016 mg/g) showed that hippocampal neurons had increased membrane damage and DNA damage [216]. A pathway analysis found a decreased synthesis of the intracellular SOD enzyme in the hippocampal neurons of these rats. Further, decreased blood serum Aβ42 levels strongly suggest irreversible toxic effects of PET-MPs because Aβ42 is a fragmentation of the amyloid precursor protein, and its reduction/absence renders neuronal injury irreversible [217].

To date, very few studies have examined the effects of MPs/NPs when exposures occur during development, and to our knowledge, no studies have evaluated transgenerational inheritance of such effects. PS- MPs/NPs (sizes 100 nm-1 μm), administered orally to female mice during pregnancy days 1 to 17, can cross the placental barrier and induce serious effects on the foetal thalamus, such as induction of ROS-mediated oxidative stress, altered GABA-ergic neurotransmission, and apoptosis, resulting in the inhibition of foetal brain development [218]. Additional studies examining other plastic types, including bioplastics, are needed and additional outcomes should be evaluated in exposed offspring, including neurobehavioral outcomes.

##### 3.2.2.3 Epidemiological Studies

An increasing body of research has identified the effects of MPs/NPs on CNS-derived cells, aquatic animals, and rodents; many of these studies also demonstrate the mechanisms of action behind these effects, including the induction of oxidative stress and inflammation, primarily through the release of ROS and lipid peroxidation, which causes DNA damage, mitochondrial dysfunction, and a decrease in neuronal networks [219, 220]. Yet, there are few studies that have evaluated the effects of long-term exposure to plastic on human health. Indeed, the use of plastic materials across sectors of the economy, including food packaging, exposes humans to a complex mixture of chemicals. With use, degradation products are formed, and the plastic itself fragments into MPs and NPs [83], which easily cross biological barriers and accumulate in various tissues [221]. The negative effects arising from the production of plastic materials, such as the increase in carbon emissions and air pollution, are already evident [222]. Ocean pollution has also attracted a lot of attention, as plastic waste damages ecosystems and enters the marine and human food chain [223]. Furthermore, many of the chemicals that are used in the production of plastics have been evaluated in human populations [224], but these studies have focused on the chemicals themselves and not MPs/NPs. To our knowledge, there is no publication that comprehensively summarizes the research conducted on how plastics are affecting human health, or which populations have been studied in this regard.

A recent report conducted on the experience of war veterans in Iraq and Afghanistan in the early years of the conflict examined the use of combustion pits to dispose of solid waste (generally 5-6% plastic, 6-7% wood, 3-4% miscellaneous, 1-2% metals and 81-84% combustible materials) [225]. This method of disposal caused an increase in emissions containing hazardous and toxic particles. Exposure to these substances has been a cause for concern in relation to respiratory diseases, cancer and neurological effects in veterans; however, no significant correlation has yet been demonstrated between exposure to combustion products and the occurrence of particular diseases in this group. A possible relationship between exposure to environmental pollutants from plastics and an increased risk of ALS has also been investigated in war veterans [226], but studies to date are also inconclusive, and the results are mixed. Although the use of burn pits during wars and conflicts makes veterans a population of concern, the incineration of waste occurs in many parts of the world including the global south. Additional studies of these populations are needed to understand whether inhalation exposures to plastics might contribute to diseases.

One recent study conducted on a population of young South African adults highlighted the lack of knowledge on the correct use of food packaging and the associated risks, in part due to the incorrect information provided by plastic identification codes, which are often ignored or misinterpreted [227]. The lack of awareness, the wrong information and often the wrong practices in the management of waste and the use of plastic material all represent a problem that is only now being addressed, thanks also to ecological movements and an increasing trend towards green life. In June 2022, the UN Environment Assembly adopted a resolution (UNEA 5/14) in which nations around the world commit themselves over the next two years to negotiate the first legally binding international treaty on plastics. To support this action, the Minderoo-Monaco Commission has been established to provide robust analyses of the health impacts of plastics and scientific solutions to protect human health [228].

## 4 PLASTIC-INDUCED EFFECTS ON THE HYPOTHALAMUS-PITUITARY-GONAD AXIS AND OUTCOMES RELEVANT TO REPRODUCTION AND FERTILITY

### 4.1 The Brain Control of Reproduction: An Overview

The hypothalamus is the brain region critical for the onset and modulation of reproduction in both sexes. Key actors in this process are the Gonadotropin-releasing hormone (GnRH) and its upstream modulator, kisspeptins [229, 230]. GnRH is a decapeptide released from the median eminence into the pituitary-portal vessels by GnRH-secreting neurons mainly located in the forebrain of non-mammalian vertebrates (*e.g*., medial anterior preoptic area (APOA)) and the mediobasal hypothalamus in mammals (*i.e*., arcuate/infundibular nucleus). In response to GnRH stimulation, pituitary gonadotrophs release gonadotropins (*i.e*., Follicle-stimulating Hormone (FSH) and Luteinizing Hormone (LH)) into the main circulation; these glycoproteic hormones reach the gonads to promote the biosynthesis of sex-steroids, as well as spermatogenesis progression in males and follicle growth and ovulation in females [230]. The physiological pulsatile release of GnRH is regulated by centrally and peripherally produced factors, primarily kisspeptin, sex steroids and metabolic sensors (*i.e*., leptin) that change in response to endogenous and exogenous environmental cues.

Because plastic particles cross the BBB, it has been hypothesized that MP/NP exposures can alter the function of cells within the hypothalamus, indirectly disrupting gonadotropins and sex steroids and, in turn, altering reproduction. Studies have also documented the direct effects of plastics on reproductive organs in both males and females. In the next paragraphs, we summarize the main studies in the field underlying the effects of MPs/NPs exposure on male and female reproduction.

### 4.2 *In vitro*, *ex vivo* and *in vivo* effects on Reproduction

#### 4.2.1 Evidence from Non-mammalian Aquatic Models: Focus on Zebrafish Model

The presence of plastic pollution in the aquatic environment is widely reported to harm a wide range of aquatic organisms and induce several types of toxicity, including reproductive toxicity due to reproductive organ tissue damage and decreased reproductive competence. For example, exposure of marine medaka (*Oryzias melastigma*) to 20 μg/L of 10 μm PS-MPs for 60 days disrupted the reproductive endocrine system and caused histological changes in the testes [231]. Effects were also observed in the development of the offspring. Similarly, a dose-dependent decrease in female fecundity was reported in Japanese medaka (*Oryzias latipes*) after 10 weeks of chronic dietary exposure to PS-MPs [232]. Moreover, PS-MPs exposures for 56 days at similar concentrations significantly decreased oocyte number, oocyte diameter and sperm velocity in oysters [233], suggesting the deleterious effects of MPs on the reproduction of numerous aquatic organisms. Because aquatic ecosystems are quickly becoming a heavily polluted environment with documented plastic wastes in virtually every locale on the planet, there are concerns for the unique and vital resources that aquatic animals rely on almost entirely for growth, reproduction and survival.

Just as they have become an invaluable resource for the study of neurotoxicity, zebrafish have also become widely used models for reproductive toxicity assessments due to their short reproductive cycles and the large number of eggs laid. In addition, the embryos are transparent, so it is fairly straightforward to observe the cell division and organ formation processes in subsequent generations following exposures to pollutants, including MPs and NPs. These zebrafish studies have revealed that MPs and NP-induced reproductive toxicity lead to alterations in fertility due to disruptions in gonad tissue integrity, as well as egg and sperm quality. For instance, a 21-day exposure to PS-MPs induced a dose-dependent impairment of the reproductive system of zebrafish [234]. At the 100 μg/L dose, a significant increase in ROS levels were found in both male and female liver and gonads, and, at the highest dose (1000 μg/L), increased apoptosis levels, histological alterations and a significant decrease in testis basement membrane thickness, were observed in male testes, suggesting disruptions to the reproductive organs. The accumulation of the PS-MPs in the gonad level was also demonstrated after 30d of exposure [159]. Similarly, high concentrations of PS-NPs resulted in oxidative stress, immunotoxicity and apoptosis in female zebrafish oocytes [235], suggesting that the female fish is also sensitive to these exposures. NPs have been shown to accumulate in zebrafish adults and can be passed on to the offspring, causing antioxidant system impairment and bradycardia without inducing other severe disturbances [236]. Direct exposure of zebrafish embryos to MPs and NPs was also shown to affect early development [237], reduce hatching and survival rates and inhibit heart rate and body length through the activation of oxidative stress and base excision repair pathways [238].

There is growing evidence that MPs act as a vehicle for a wide range of co-contaminants. Some indirect effects of MPs-induced toxicity are linked to these contaminants, which are adsorbed on their surface. For example, a 60d study examining co-exposure of PS-MPs and microcystin-LR (MC-LR), reported enhanced accumulation and exacerbated deleterious effects of the MC-LR in zebrafish gonads [239]. In particular, seminiferous epithelium deterioration and widened intercellular spaces were observed in the testis, and basal membrane disintegration and zona pellucida invagination were found in the ovary. Moreover, genes relevant to the hypothalamic-pituitary-gonadal axis function were altered. Furthermore, in another co-culture study, the chronic co-exposure of zebrafish to benzo[α]pyrene (BaP)-contaminated PE-MPs, revealed impaired reproductive performance and disrupted bone mineralization in offspring larvae [240]. The toxic effects of EDCs such as bisphenols have also been assessed in the zebrafish model, suggesting their multiorgan toxicity [241-243]. Co-exposures to bisphenol F and NPs demonstrated that the co-exposure decreased the number of eggs laid, impaired the locomotor behavior of parental zebrafish, and impacted the hatching rate, mortality, body length and locomotor behavior of offspring zebrafish [244].

#### 4.2.2 Evidence from Mammalian Models

Some of the first studies to detect MPs in human tissues revealed the presence of these particles in the placenta [18, 90, 245, 246]. Very recently, MPs/NPs were detected in human testis (PS) and semen (PE and PVC), providing basic data for the risk assessment of MPs to human reproductive health [247]. In rodents, MPs/NPs can enter ovarian granulosa cells [248], and testicular Sertoli and Leydig cells [249], providing evidence that the gonad is a direct target of plastic particles. Because documented internal exposures are relatively new, few studies have examined the consequences of such exposures on the reproductive organs. Bioaccumulation in the gonads has now been demonstrated, with the ovary more sensitive than the testis [250]; hormonal imbalances, testicular damage at both germ cells and intra-gonadic somatic cells, poor sperm quality, ovarian cysts, granulosa cell death, reduced follicular growth, and reduced pregnancy rates are all endpoints that have been shown to be the main consequences of exposures [6, 251-253].

##### 4.2.2.1 Effects on Female Reproduction

Most studies *in vitro* or in rodents that have evaluated the effects of MPs/NPs on female reproductive outcomes have focused on the ovary, including disruptions to the cell populations in the ovary, the morphology of ovarian follicles, or in the expression of genes involved in ovarian function [251]. For example, mice exposed to PS-MPs for 35d had irregularly shaped follicles with loosely arranged granulosa cells, fewer healthy follicles, and an increase in atretic follicles [254]. Transmission electron microscopy approaches confirmed that PS-MPs exposures increased mitochondrial swelling and nuclear enlargement, contributing to organelle fragmentation and eventual apoptosis of the granulosa cell layer. Additional studies have confirmed the effects of PS-MPs on the number of antral follicles in the ovary following a 35d exposure period, although effects on other follicle types were not observed [255]. Like what was observed in several studies examining CNS structures, exposures to PS-MPs increased inflammatory cytokines in the ovary, although markers of ROS were not altered. Similar outcomes were observed in female mice administered PS-NPs, where oral exposures for 35d reduced the number of growing follicles and lowered serum concentrations of anti-mullerian hormone compared to control mice [256]. Again, PS-NPs exposures were shown to increase apoptosis in granulosa cells in the ovary; the expression of pro-apoptotic genes was also upregulated in the ovary, whereas the apoptosis-inhibiting gene, *Bcl-2*, was significantly downregulated. Other female reproductive organs that have been examined following exposures to MPs include the oviducts, which were dilatated in female mice administered PE-MPs in adulthood prior to and throughout pregnancy and lactation [257].

When fertility outcomes themselves are considered, mice administered PE-MPs during pregnancy and lactation had no effect on the length of the gestation period, but the number of live births per dam and the average body weight of the pups was decreased in litters born to dams administered 2 mg/day [257]. In contrast, mice administered PS-NPs had significantly diminished fertility (a 50% reduction in the ability to become pregnant) but no difference in gestation length or litter size [256]. Furthermore, a study that evaluated oocytes that were removed from females exposed to PS-MPs and then super-ovulated found no differences in the number of recovered oocytes but a decrease in the number of oocytes that survived through *in vitro* fertilization, suggesting that microplastic exposures affect the maturation and survival of oocytes [255]. Because fertility is dependent on the proper functioning of the hypothalamic-pituitary-gonadal axis, it is not currently clear whether these effects are due to the direct impact on the ovary or whether the brain is also mechanistically implicated in these outcomes. Furthermore, outcomes like oestrous cyclicity, the timing of puberty, and measures of reproductive aging - all of which are dependent on a functional hypothalamic-pituitary-ovarian axis - have not yet been evaluated in females exposed to MPs/NPs.

##### 4.2.2.2 Effects on Male Reproduction

Although the male reproductive tract includes several organs that function together to support sperm production, sperm maturation and semen quality, most studies of MPs/NPs have focused solely on aspects of testicular health. Exposures to PS-MPs have been shown to compromise serum concentrations of testosterone [9] (for recent review); they also induce oxidative stress, mitochondrial disfunction, apoptosis, and inflammation [246, 247, 258-265] and ultimately impair spermatogenesis due to disruption of the blood-testis-barrier (BTB) [265, 266]. The BTB is critical for the maintenance of a suitable microenvironment for the preservation of mitotic/pre-meiotic stages (*i.e*., basal compartment) and the formation of post-meiotic stages and spermiogenesis (*i.e*., luminal compartment) [267]. Importantly, these effects can be drastic when exposures occur during development. For example, following prenatal and postnatal exposure to PS-MPs in rodents, key processes for the physiology of testis development and spermatogenesis were disrupted, including urogenital system development, formation of the primary germ layer, histone methylation, hormone biosynthesis and sex hormone signaling pathways [247].

A recent study focused on the effects of NPs on mouse spermatozoa [268]. Administration of PS-NPs *in vivo* was shown to bypass the epididymal microstructure to enter the spermatozoa and accumulate in its head and midpiece. As a consequence, the spermatozoa failed to undergo either spontaneous or induced capacitation or capacitation-related processes (*e.g*., spontaneous/induced acrosome reaction, hyperactivation, flagellum waveform *etc*.). Several factors that are key to sperm capacitation were disrupted in PS-NP-exposed spermatozoa, including reduced levels of F-actin polymerization.

Both *in vivo* studies and *in vitro* (*i.e*., TM4 Sertoli cells) models have been used to investigate the molecular mechanisms responsible for the effects of MPs/NPs on the testis. These studies have revealed the occurrence of oxidative stress and cytoskeleton disorganization. At the molecular level, the truncation of actin filaments due to the differential expression of the actin-binding proteins [258] and increased degradation of the junctional proteins in the BTB [269] have been demonstrated. Furthermore, endoplasmic reticulum stress is induced in the testis of MPs-treated mice, along with upregulation of apoptotic genes in the testis [246]. RNA-seq screening has similarly revealed differentially expressed genes related to cell junctions, cytoskeleton, and oxidative stress following treatments with PS-MPs [258].

The large hydrophobic surface of MPs makes them a suitable vehicle to carry out several environmental pollutants like endocrine disruptors or heavy metals, among others [6, 8]. Hence, several *in vivo* studies reported more harmful and additive effects on male reproduction than the environmental toxicant alone in the case of co-administration of MPs [270-273].

Similarly, investigations on NPs confirmed that these particles can aggravate the toxic effects of chemicals such as plastic additives like phthalates. In this respect, a study reported the additive toxic reproductive effects of PS-NPs and the phthalate DEHP on spermatogenesis; although pituitary gonadotropins and sex steroids were not significantly affected by DEHP-contaminated MP-NPs, the combined treatment caused the production of poor quality spermatozoa [273]. Transcriptomic analysis was then used to identify a large set of deregulated intratesticular targets related to immune response, oxidative stress, cell signaling, protein ubiquitination, cell death and mitochondrial physiology.

Since the release of MPs in the environment alters their physical and chemical properties and causes their fragmentation into NPs, an additional open question is how the effects of aged plastic might differ from the effects of pristine plastics. A recent study compared aged *vs.* pristine MPs and revealed that 1-week intratracheal exposures to aged PS-MPs (diameter of 4-5 µm) significantly altered biochemical indicators in serum with toxic effects that were higher in liver and spleen than testis, but with consequences on metabolism, immune functions and spermatogenesis [274].

Unlike the female, who is born with a full complement of oocytes and can never produce more, males produce sperm throughout their lives. Thus, an intriguing question is the degree to which MPs-induced damage is reversible. To address this question, Liu and coworkers [275] first reported the effects of 5 µm PS-MPs administered in drinking water, including mitochondrial damage, which was shown to be the main target of oxidative stress causing spermatogenesis failure; then, the animals were allowed to recover from exposures for a long period lasting 1-2 spermatogenetic cycles. The authors demonstrated the possible rescue of spermatogenesis [275]. Additional studies have evaluated whether the administration of natural products with antioxidant properties like the flavonoid astilbin [276], pinostrobin [277], rhamnetin [278] or ROS scavengers N-Acetylcisteine [262] exerts protective effects against MPs toxicity on pituitary gonadotropins, sex steroid production, oxidative stress, inflammation, cell damage, spermatogenesis failure and sperm quality [276]. These findings suggest that MPs do not affect the pool of intratesticular stem germ cells; in fact, their effects seem restricted to the exposure time and may be counteracted by dietary antioxidants, as also occurs for other plasticizers acting as endocrine disruptors [279].

## CONCLUSION

Plastics in the micrometer and nanometer range are contaminants of emerging concern for all ecosystems. Most eco-toxicological studies have been carried out on aquatic species, particularly marine species, and the knowledge on the impact of MPs in terrestrial animals, including mammals and humans, is still in its infancy. In addition, one of the major limitations of MPs/NPs-related research in environmental science and toxicity is that the surrogate materials employed in the studies are idealized and often present very regular shapes (spheres or fibres). Such materials are not representative of MPs occurring in real systems, in which plastic debris different in size, shape, or composition interact with each other or with other environmental contaminants. It has been widely demonstrated that, individually, the different plastic types/sizes/shapes and co-contaminants may induce different kinds of toxicities in zebrafish due to the different mechanisms of absorption, penetration, accumulation and contaminant release at diverse tissue levels [16]. Therefore, it would be desirable that future studies will be carried out in conditions as close as possible to the real situation. Nevertheless, upcoming data from *in vitro* cell models and mammals - albeit in a possible scenario in terms of doses, exposure route and duration- strongly suggest these contaminants may heavily impact human health due to their ability to bypass biological barriers like the BBB, BTB, or placental barrier, to circulate in the blood and to accumulate in secondary tissues. In such a way PLATAMINATION poses the conditions for brain disease and reproductive failure as a consequence of neurotoxicity, microglia activation and neuroinflammation in different brain areas, including the hypothalamus that controls reproduction. MPs and NPs may also induce adverse effects on offspring, posing health risks by trans-generational inheritance. In fact, epigenetic factors, including stress, environmental pollutants and alterations of gut microbiota, collectively defined as the “exposome,” have been suggested to cause pathological imprinting of the immune system [280, 281]. The imbalance in cytokine production in the foetal brain might be responsible for an abnormal microglial and astrocyte signature leading to increased susceptibility to neurological diseases.

The recent detection of plastic debris in human stool, placenta, semen or blood [7, 18, 245, 282-284] provided the first qualitative and quantitative evidence of MPs exposure in humans to address further studies in the field. In this respect, there is the need to check for MPs/NPs in biological tissues, particularly in mammals and humans, to pose the basis for *ex vivo*, *in vitro,* and *in vivo* investigations at realistic doses and with environmentally relevant plastic types.

Furthermore, the heterogeneous composition of plastic debris in terms of chemical composition, properties or size makes them a suitable vehicle to concentrate and carry out in tissues different and well-known environmental contaminants like endocrine disruptors or heavy metals notably capable of interfering in brain homeostasis and physiology. At present, there have been no suggestions that MPs should be considered endocrine-disrupting chemicals (EDCs), but it should be noted that MPs and NPs display several of the key characteristics of EDCs [285], including alterations to circulating hormone concentrations and effects on hormone-responding organs. The use of plastic mixtures that mimic the size and types of plastics in the environment alone or in combination with well-known ECDs may be relevant to studying the molecular mechanisms related to plastic toxicity and direct/indirect ECD activity. However, the introduction in the market of bio-degradable plastics may have a positive impact on the environment, but currently does not ensure their safety due to the paucity of studies in the field.

Hence, the neurological and reproductive health risks of MPs/NPs exposure warrant serious consideration and further studies on biodegradable plastics are recommended.

## Figures and Tables

**Fig. (1):**
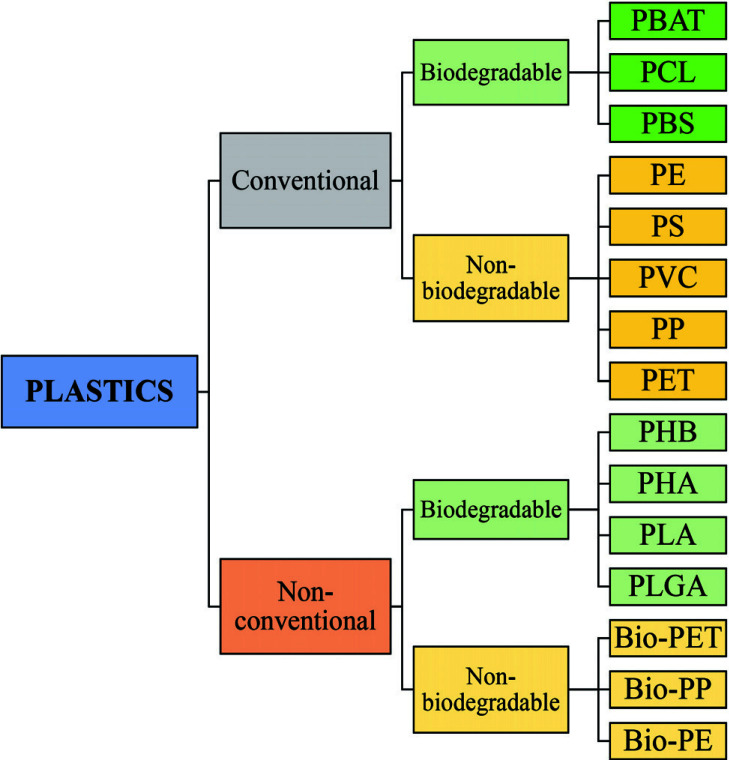
Classification of plastic materials. **Abbreviations:** Bio-PE, bio-polyethylene; Bio-PET, bio-polyethylene terephthalate; Bio-PP, bio-polypropylene; PBAT, polybutylene adipate terephthalate; PBS, polybutylene succinate; PCL, polycaprolactone; PE, polyethylene; PET, polyethylene terephthalate; PHA, polyhydroxyalkanoates; PHB, polyhydroxybutyrate; PLA, polylactic acid; PLGA, polylactic co-glycolic acid; PP, polypropylene; PS, polystyrene; PVC, polyvinyl chloride.

**Fig. (2):**
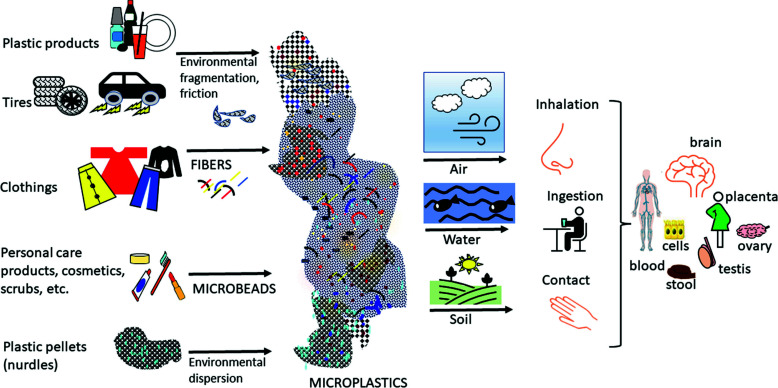
PLASTAMINATION: Plastic sources, types, diffusion, exposure routes for living organisms, humans included, accumulation in tissues and excretion. The diffusion of plastic debris from different sources in air, water or soil causes their entry into living organisms by inhalation, ingestion or contact, respectively. Once in the body, these substances bypass the biological barriers and enter into biological fluids and tissues, thus causing inflammation and toxic effects; when ingested, plastic debris is released in the stool.

**Table 1:** Chemical characteristics and uses of the most diffused conventional and non-conventional plastics.

**Polymer**	**Chemical Structure**	**Main Chemical Properties**	**Applications**	**References**
PE	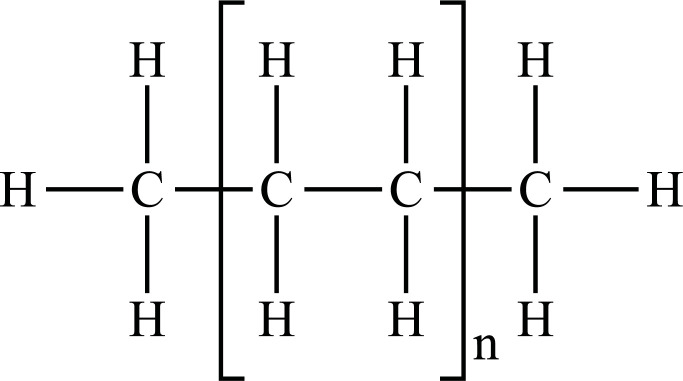	**Melting point:** 92°C **Density:** 0.962 g/mL at 25°C **Solubility:** acetone and benzene. Insoluble in water. **Degradation ability:** stable, but it breaks down under UV light or sunlight over two years in the environment.	Dispensing bottles, wash bottles, tubing, plastic bags, protective coatings on paper, textiles, cling film, carrier bags, agricultural film, milk carton coatings, electrical cable coating, and heavy-duty industrial bags.	[32-34]
PP	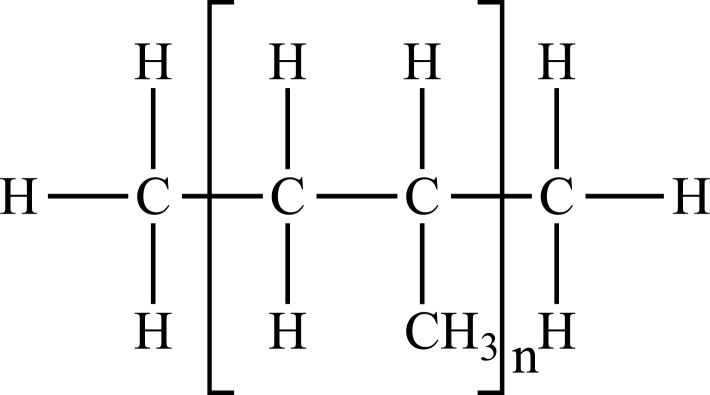	**Melting point:** 157°C **Density**: 0.9 g/mL at 25°C **Solubility:** insoluble in cold organic solvents and water **Degradation ability:** resistant to photo- and thermal oxidation at modest temperatures. It can be degraded by *Aspergillus niger* and bacteria such as genera *Vibrio* and *Pseudomonas*. UV-irradiation, thermal treatment, and gamma-irradiation pretreatment methods make it more susceptible to degradation.	Bottles, pails, fibres, film sheets, instruments, syringes, pouches, hospital disposables, test tubes, beakers, pipettes, synthetic suture material.	[32, 35-37]
PVC	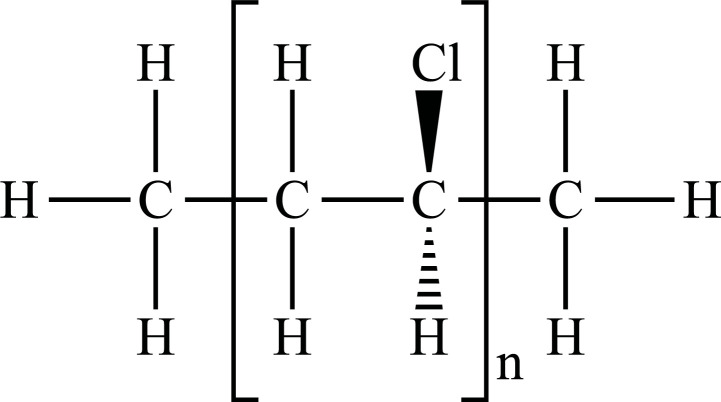	**Melting point:** 170-195°C **Density**: 1.4 g/mL at 25°C **Solubility:** tetrahydrofuran. Insoluble in water and alcohol. **Degradation ability:** stable. It can be degraded and recycled by cracking/pyrolysis. Degradable through three stages: T<250°C, dehydrochlorination to polyene; 250°C<T<350°C, polyene decomposes to low-molecular-weight compounds; 350°C<T, polyene breaks down into low-molecular-weight compounds. PVC ingested by *Tenebrio molitor* larvae depolymerizes within 12-15 h.	Bottles, blister packs, transparent packs and punnets, safety equipment, insulation on pipes, jacketing, electricity distribution boxes, switches, transparent distributor box housings, credit cards, and traffic signs.	[32, 38-41]
PS	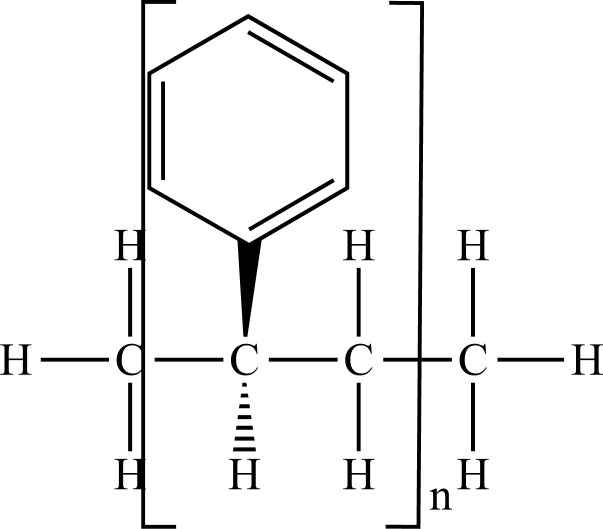	**Melting point:** 212°C **Density**: 1.06 g/mL at 25°C **Solubility:** chloroform. **Degradation ability: s**table, resistant to biodegradation, and susceptible to photo-oxidation. Degradable and recyclable through thermolysis or chemical treatment combined with pyrolysis.	CDs, toys, cosmetic products, food containers, trays, plates, and cups, packaging products, toys, clips, office supplies, and car tires.	[32, 42, 43]
PET/ Bio-PET	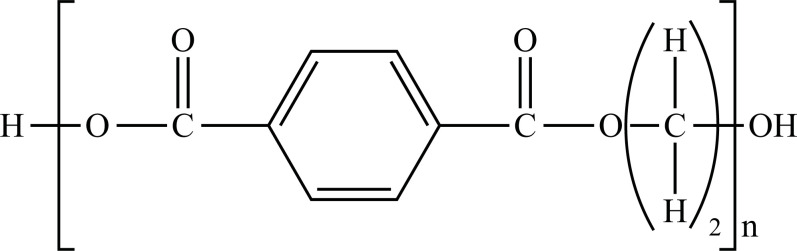	**Melting point:** 250-255°C **Density**: 1.68 g/mL at 25°C **Solubility:** trifluoroacetic acid, hexafluoro isopropanol. **Degradation ability:** degradable and recyclable through solvent decomposition and pyrolysis. Highly durable and robust, with an estimated half-life of over 2500 years. It undergoes degradation into microplastics under UV/heat in marine ecosystems. Aerobic biodegradation is allowed by PETase of *Ideonella sakaiensis*, *Thermobifida*, *Saccharomonospora* and *Streptomyces*.	Containers for beverages, food industry, packaging trays, blister, cosmetic jars, microwave containers, agriculture applications, automotive and textiles Construction, transport and packaging sector, textiles industry, agriculture/horticulture, flexible and rigid packaging.	[32, 44-48]
PBAT	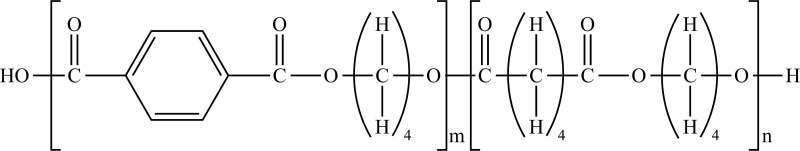	**Melting point:** 120°C **Density**: 1.26 g/cm^3^ **Solubility:** dichloromethane, hexafluoroisopropanol, and tetrahydrofuran. **Degradation ability:** degraded into carbon dioxide, water, and other small molecules under soil and compost conditions with the influences of heat, water, oxygen, enzymes, and organisms. PBAT-degrading microorganisms includes *Sphingopyxis ginsengisoli*, *Bacillus pumilus*, *Pseudomonas pseudoalcaligenes*, *Cryptococcus*, and *Trichoderma asperellum*	Mulching films, compostable bags, nonwoven sheets and textiles, catering goods, foams for food packaging, courier bags, cutlery, cling wrap, paper cups, agriculture, fishery, forestry, and textiles scaffolds for tissue engineering.	[49-53]
PCL	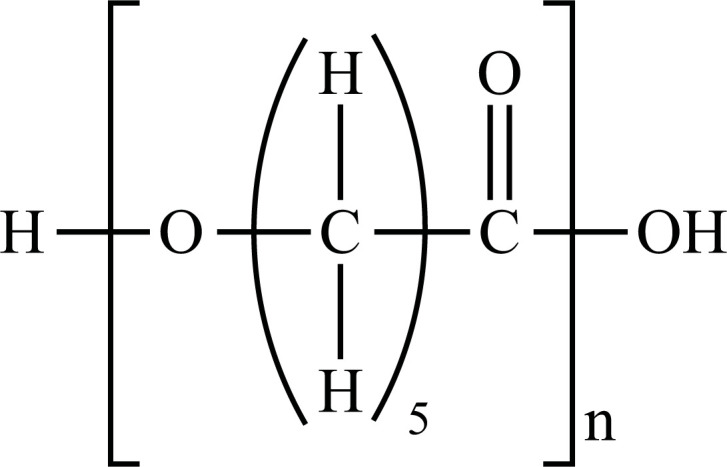	**Melting point:** 60°C **Density**: 1.146 g/mL at 25°C **Solubility:** chloroform, dichloromethane, and tetrahydrofuran. **Degradation ability:** degraded by hydrolytic process. Degradation occurs in biotic environments, including soil, seawater, active sludge and compost. Biodegradation can occur by several fungi, such as *Penicillium funiculosum, Aspergillus flavus, Rhizopus delemar, R. arrizus,* and *Candida cylindracea* and bacteria such as *Tenacibaculum, Alcanivorax, Pseudomonas, Alcaligenes faecalis, Bacillus pumilus,* and *Clostridium acetobutylicum*.	Medical applications such as implantable drug delivery systems, drug carriers, vaccine carriers, tissue engineering applications, and 3D scaffolds.	[32, 54-57]
PBS	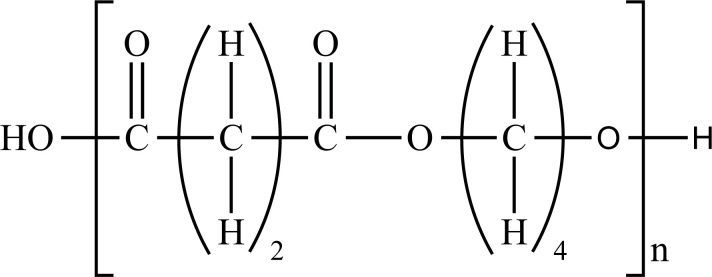	**Melting point:** 115°C **Density**: 1.26 g/cm^3^ **Solubility:** chloroform, insoluble in water. **Degradation ability:** degraded into water and carbon dioxide by hydrolytic or enzymatic degradation. Microbial biodegradation can occur through *Pseudomonas cepacia* and at 30°C through *Terribacillus sp.* JY49.	Food packaging, mulch film, plant pots, hygiene products, fishing nets, fishing lines.	[58-61]
PLA	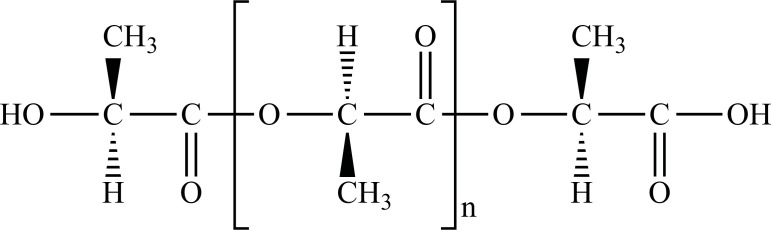	**Melting point:** 262°C **Density**: 1.25-1.28 g/cm^3^ **Solubility:** dioxane, acetonitrile, chloroform, and methylene chloride **Degradation ability:** degradation through hydrolytic, oxidative, thermal, and microbial, enzymatic, and photodegradative processes. The human body degrades PLA through hydrolysis of the ester-bond backbone. In the environment is chemically hydrolyzed into low molecular weight oligomers and then mineralized into carbon dioxide and water by existing microorganisms such as *Stenotrophomonas pavanii* and *Pseudomonas geniculate.*	Medical implants and devices, fibres (carpet, clothing), packaging, cups, food containers, trays, cutlery, salad bowls, straws, tea bags, coffee pods, flexible packaging film, and bottles.	[32, 62-65]
PLGA	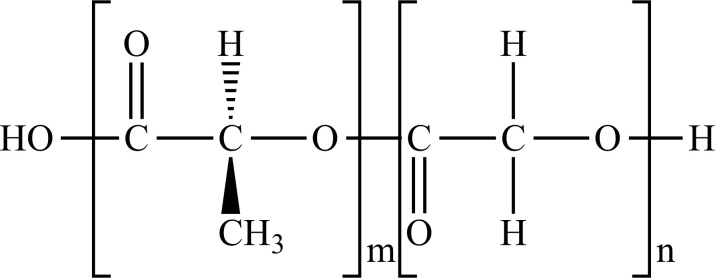	**Melting point:** 262°C **Density**: 1.53 g/mL at 25°C **Solubility:** tetrahydrofuran, acetone, and ethyl acetate. **Degradation ability:** completely biodegradable inside the body. In aquatic environments, PLGA degrades *via* hydrolysis, breaking down into water-soluble fragments. Hydrolytic enzymes such as lipases from *Candida antarctica*, *Candida cylindracea*, *Candida rugosa*, *Mucor miehei, Rhizopus arrhizus* and the esterase from *M. miehei,* favor PLGA biodegradation.	Endodontic treatments, periodontal treatments, implant therapy, dentin regeneration, vaccine carriers, drug carriers, and bone regeneration.	[66-69]
PHA	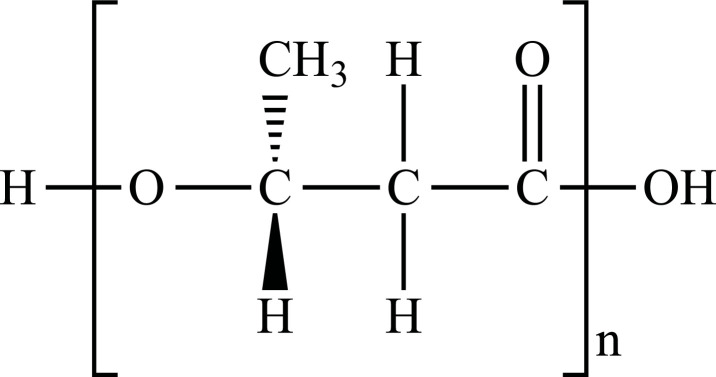	**Melting point:** 180°C **Density**: 1.25 g/cm^3^ **Solubility:** chloroform and insoluble in water. **Degradation ability**: degradation occurs in environments, including soil, lake water, marine water, and sewage sludge by the genera *Mitsuaria*, *Chitinophaga*, and *Acidovorax* and fungi such as *Penicillium, Fusarium*.	Straws, cups, lids, bottles, produce bags, shopping bags, utensils, diaper linings, plates, wipes, toys, trash bags, seals, labels, glues, and drug carriers.	[70-75]

**Abbreviations:** Polyethylene (PE); Polypropylene (PP); Polyvinyl chloride (PVC); Polystyrene (PS); Polyethylene terephthalate (PET); Bio-polyethylene terephthalate (Bio-PET); Polybutylene adipate terephthalate (PBAT); Polycaprolactone (PCL); Polybutylene succinate (PBS); Polylactic acid (PLA); Polylactic co-glycolic acid (PLGA); Polyhydroxyalkanoates (PHA).

**Table 2:** Overview of studies reporting neurotoxic effects of micro- and nanoplastics on zebrafish (*Danio rerio*).

**Neurotoxic Effects**	**Characteristics of Plastic Particles**	**Co-contaminants**	**Plastic Particle Size**	**Plastic Particle Concentration**	**Exposure Time**	**References**
**(NPs**): • Altered locomotor activity; • Hypoactivity; • Reduced larvae body length; • Acetylcholinesterase activity inhibition by 40%.	PS-MPs and NPs	None	MPs: 45 μm NPs: 50 nm	1 mg/L	120 hpf	[180]
• Alterations of microbiome; • Altered energy, nucleic acid and glycolipid metabolic profiles; • Inflammatory response; • Neurotoxic response; • Oxidative stress.	Fluorescent PS-MPs	None	5 and 50 μm	100 and 1000 μg/L	7 dpf	[181]
• Abnormal behavior (*i.e*., seizures and tail bent downward); • Effects on the aryl hydrocarbon receptor (AHR) pathway; • Disruption of the oogenesis process.	PE-MPs	None	10-22 μm, 45-53 μm, 90-106 μm, 212-250 μm, and 500-600 μm	2 mg/L	96 hpf	[182]
• Altered neurotransmitter levels (DA, ACh, 5-HT, MT, GABA); • Impaired locomotor activity; • Aggressiveness; • Shoal formation; • Predator avoidance behavior; • Dysregulated circadian rhythm; • Oxidative stress; • Lipid and energy metabolism alterations.	FITC-conjugated PS-NPs	None	~70 nm	0.5-1.5 ppm	7 days	[159]
• ROS induction; • Antioxidant defense system disruption; • Neurotransmission impairment; • Histopathological lesions, including inflammation, degeneration, necrosis, and hemorrhage in the brain and liver tissues; • Upregulation of *gstp1*, *hsp70l* and *ptgs2a* genes along with the downregulation of *cat*, *sod1*, *gpx1a* and *ache* genes.	PS-MPs	None	100 nm	10, 100 µg/L	35 days	[164]
• Inhibition of AChE activity; • Impaired swimming competence; • Affected avoidance behavior.	Red opaque fluorescent polymer microspheres	Copper (60 and 125 µg/L)	1-5 µm	2 mg/L	14 days	[183, 184, 184]
• Oxidative stress; • Neurotoxicity; • Increased respiratory rate; • Disturbed energy metabolism; • Inflammatory response.	PE-MPs	None	45-53 μm	0.6 mg/L	1, 5 and 10 days	[185]
• Inflammatory response; • Activation of xenobiotic metabolism; • Acetylcholinesterase activity inhibition.	PS- and high-density PE-MPs	None	90, 50 and 25 μm	100 μg/L	20 days	[186]
• Oxidative stress; • Decreased antioxidant defence; • Increased acetylcholinesterase activity; • Numerical changes in neuroblasts.	Naturally-aged PS- MPs	None	~ 17 μm	4×10^4^ and 4×10^6^ microparticles/m^3^	5 days	[187]
**Co-exposure MPs/TCS:** • Oxidative stress and lipid peroxidation in the liver; • Enhanced neurotoxicity in the brain.	PE, PP and PVC-MPs	Triclosan (TCS) (300 μg/L)	1-15 μm	200 mg/L	28 days	[188]
• **NPs:** Severe neurotoxicity and oxidative stress; • NPs-9-NAnt complex reduced the biological toxicity of zebrafish with respect to NPs alone.	PE-MPs	9-Nitroanthracene (9-NAnt), (5 and 500 μg/L)	100-150 μm	10 and 40 mg/L	4, 7 and 21 days	[179]
• Behavioral changes (in shoal); • Increased acetylcholinesterase activity; • REDOX imbalance; • Changes in zebrafish surface pigmentation.	PLA-MPs	None	2.34 ± 0.07 μm	2.5 and 5 mg/L	30 days	[176]
**Co-exposure NPs/KCZ/FCZ:** • Induction of ROS production, • Oxidative stress; • Increased apoptosis; • Reduced hatching and survival rate; • Developmental toxicity	Fluorescent PS-NPs	Ketoconazole (KCZ) and Fluconazole (FCZ)	0.05 μm	1 mg/L	96hpf	[189]
**Co-exposure NPs/MTM:** • Oxidative stress (increased ratio GSH/GSSG, upregulation of oxidative stress-related genes, such as *sod, sod mt* and *gpx4a*); • Altered metabolites are involved in several pathways, such as Arachidonic acid metabolism, Biotin metabolism, Pyruvate metabolism, Glutathione metabolism, and Porphyrin and chlorophyll metabolism; • Altered neurotransmitter-related genes (*ache, gfap* and *scl1A3b*); • Impaired locomotor activity.	Fluorescein isothiocyanate (FITC)-conjugated PS-MPs	Methylmercury (MTM), (1 μg/L)	5 μm	1 mg/L	7 days	[190]
**Co-exposure NPs/AVO:** • Altered genes related to the nervous system (axons, dendrites, synapses, axon myelin sheath formation) and retinal system development; • Acetylcholinesterase activity increases; • Swimming behavior alterations • Oxidative stress (SOD, CAT).	fluorescent PS-NPs	Avobenzone (AVO) (10 μg/L)	100 nm	10 μg/L	144 hpf	[191]
• Downregulation of neuronal proliferation (sox2, pcna), neurogenesis (neuroD, olig2) and motor neuron development (islet) related genes.	Red opaque fluorescent polymer microspheres	Copper (60 and 125 µg/L)	1-5 µm	2 mg/L	14 dpf	[192]
• Oxidative stress; • Dysregulation of serotonin synthesis and apoptosis pathways.	Red opaque fluorescent polymer microspheres	Copper (25 µg/L)	1-5 µm	2 mg/L	30 days	[193]
• Hypoactivity; • Seizure-like behavior; • Dysregulation of cholinergic, dopaminergic and GABAergic systems.	PS-MPs	None	1, 6, 10 and 25 μm	500, 5000 and 50000 particles/mL	120 hpf	[194]
• Abnormal hyperactive swimming behavior; • Obvious nervous system interference; • Altered neurotransmitter levels.	PE-MPs	None	40-47 μm	0.1-10 mg/L	7 days	[161]
• Acetylcholinesterase activity inhibition; • Altered CYP450 induction; • Modified behavioral patterns.	50% PE, 25% PP, 15% PS and 10% PVC- MPs	Several environmental co-contaminants are present in natural conditions (pharmaceuticals, pesticides, additives, and sweeteners)	< 50 μm	100 mg/L	21 days	[195]
• Charge-specific neurotoxicity with consequent behavioral differences; NH2-modified NPs induced stronger developmental toxicity (decreased spontaneous movement, heartbeat, hatching rate and length) and more serious cell apoptosis in the brain inducing greater neurobehavioral impairment; • Charge-specific metabolite alteration; NH2-modified NPs decreased levels of glycine, cysteine, glutathione, and glutamic acid; COOH-modified NPS increased levels of spermine, spermidine, and tyramine.	differently charged PS-Ps - positive charge: -NH_2_-modified; - negative charge: -COOH-modified	None	50 nm	30 and 50 mg/L	120 hpf	[165]
• Intestinal inflammation; • growth inhibition; • restricted development; • disrupted regulation within the brain- the intestine-microbiota axis due to microbiome and neurotransmission alterations.	PS-NPs	None	44 nm	1, 10, 100 μg/L	30 days	[174]
**Co-exposure NPs/4-NP:** • Oxidative damage (reduced CAT activity and GSH level); • Energy metabolism disruption (a-KGDH activity); • Neural cell death; • Acetilcholinesterase and glutamine synthetase activities inhibition; • Glutamate dehydrogenase activity increase.	PS-NPs	Nonylphenol (4-NP) (1 μg/L)	20-80 nm	0.1, 1, 10 and 100 μg/L	45 days	[196]
• Neurobehavioral alterations; • Altered neurotransmitter levels (DA, GABA, 5-HT, and ACh)	Rodhamine B-labeled UV-aged PS-MPs	None	1 μm	0.1-100 μg/L	120 hpf	[160]
• Oxidative stress (altered SOD, CAT, GST and MDA); • Altered neurotransmitter levels (5-HT, GABA, DA, and ACh); • Altered cholinergic system (AChE, ChAT, ChE) activity; • Impaired locomotor activity	UV-aged PS-MPs	None	10 μm	100 μg/L	96 hpf	[163]
**Co-exposure NPs-AS:** • Oxidative stress; • Mitochondrial damage; • Histopathological alterations in the brain; • Altered DA and Ach metabolism.	PS-NPs	Arsenic (2.83-5 mg/L)	100 nm	1 mg/L	30 days	[197]
• Increased oxidative stress genes *(SOD 1*, *SOD 2)*; • Down-regulated antiapoptotic genes (*hsp70*, *Bcl2a)*; • Inflammatory and apoptosis response (*cas1*, *cas8* and *IL1β);* • Acetylcholinesterase activity inhibition; • Down-regulated DNA repair genes (gadd45α and rad51).	PS-NPs	None	30 nm	0.1, 0.5 and 3 ppm	120 h	[198]
• Neuronal loss, axonal deletion/shortening/hybridization; • Developmental and apoptotic-related gene alterations; • Behavioral abnormalities • Altered GABAergic, cholinergic and serotonergic systems.	Fluorescent PS-NPs	None	100 nm, 500 nm, 1000 nm	8.6 mg/L	120 hpf	[199]
• Acetylcholinesterase activity inhibition; • Altered endocrine-related gene expression profiles both in the thyroid and glucocorticoid axes; • Altered behaviors: increased activity and anxiety at lower doses and lethargy at higher doses.	Fluorescent polystyrene NPs	None	30 nm	0.1, 0.5 and 3 mg/L	120 hpf	[200]
**Co-exposure NPs/BDE-47:** • Neurocentral development markers *ache* and *chrn7α* downregulation;	PS-NPs	Persistent organic pollutants (POPs) such as polybrominated diphenyl ethers (PBDEs): 2,2',4,4'-tetrabro-modiphenyl ether (BDE-47), (0,1, 10 μg/L)	80 nm	0.05-10 mg/L	120 hpf	[201]
• Developmental stunting • Decreased survival and hatching rates; • Reduced voluntary locomotion; • Induced anxiety-like behaviors; • Affected brain-derived neurotrophic factor (BDNF): affected circadian behavior.	PLGA- and PLA- MPs	None	PLGA: 472.5-4213.5 nm PLA: 667.5- 4213.5 nm	1, 25, 50, 100, 250, and 500 mg/L	96 hpf	[175]

## LIST OF ABBREVIATIONS

ACh: Acetylcholine
AchE: Acetylcholinesterase
ALS: Amyotrophic Lateral Sclerosis
Aβ: Amyloid‐β
BBB: Blood-brain Barrier
BTB: Blood-testis-barrier
CNS: Central Nervous System
CSF: Cerebrospinal Fluid
DA: Dopamine
DG: Dentate Gyrus
GFAP: Glial Fibrillary Acidic Protein
ISF: Interstitial Fluid
MPs: Microplastics
MS: Multiple Sclerosis
NIAS: Non-intentionally Added Substances
NPs: Nanoplastics
PE: Polyethylene
PP: Polypropylene
PS: Polystyrene
PVC: Polyvinyl Chloride
TBI: Traumatic Brain Injury
TGF: Transforming Growth Factor
TNF-α: Tumor Necrosis Factor-α
VEGF: Vascular Endothelial Growth Factor

## References

[1] Jiang B., Kauffman A.E., Li L., McFee W., Cai B., Weinstein J., Lead J.R., Chatterjee S., Scott G.I., Xiao S. (2020). Health impacts of environmental contamination of micro- and nanoplastics: A review.. Environ. Health Prev. Med..

[2] Schmid C., Cozzarini L., Zambello E. (2021). Microplastic’s story.. Mar. Pollut. Bull..

[3] Bajt O. (2021). From plastics to microplastics and organisms.. FEBS Open Bio.

[4] Jin M., Wang X., Ren T., Wang J., Shan J. (2021). Microplastics contamination in food and beverages: Direct exposure to humans.. J. Food Sci..

[5] Blackburn K., Green D. (2022). The potential effects of microplastics on human health: What is known and what is unknown.. Ambio.

[6] D’Angelo S., Meccariello R. (2021). Microplastics: A threat for male fertility.. Int. J. Environ. Res. Public Health.

[7] Zhang Q., He Y., Cheng R., Li Q., Qian Z., Lin X. (2022). Recent advances in toxicological research and potential health impact of microplastics and nanoplastics *in vivo*.. Environ. Sci. Pollut. Res. Int..

[8] Maradonna F., Meccariello R. (2023). EDCs: Focus on reproductive alterations in mammalian and nonmammalian models.. Environmental Contaminants and Endocrine Health..

[9] Ullah S., Ahmad S., Guo X., Ullah S., Ullah S., Nabi G., Wanghe K. (2023). A review of the endocrine disrupting effects of micro and nano plastic and their associated chemicals in mammals.. Front. Endocrinol..

[10] Viršek M.K., Lovšin M.N., Koren Š., Kržan A., Peterlin M. (2017). Microplastics as a vector for the transport of the bacterial fish pathogen species Aeromonas salmonicida.. Mar. Pollut. Bull..

[11] Ma C., Chen Q., Li J., Li B., Liang W., Su L., Shi H. (2021). Distribution and translocation of micro- and nanoplastics in fish.. Crit. Rev. Toxicol..

[12] Wang W., Ge J., Yu X. (2020). Bioavailability and toxicity of microplastics to fish species: A review.. Ecotoxicol. Environ. Saf..

[13] Xu S., Ma J., Ji R., Pan K., Miao A.J. (2020). Microplastics in aquatic environments: Occurrence, accumulation, and biological effects.. Sci. Total Environ..

[14] Deidda I., Russo R., Bonaventura R., Costa C., Zito F., Lampiasi N. (2021). Neurotoxicity in marine invertebrates: An update.. Biology.

[15] Yong C., Valiyaveettil S., Tang B. (2020). Toxicity of microplastics and nanoplastics in mammalian systems.. Int. J. Environ. Res. Public Health.

[16] Bhagat J., Zang L., Nishimura N., Shimada Y. (2020). Zebrafish: An emerging model to study microplastic and nanoplastic toxicity.. Sci. Total Environ..

[17] Windheim J., Colombo L., Battajni N.C., Russo L., Cagnotto A., Diomede L., Bigini P., Vismara E., Fiumara F., Gabbrielli S., Gautieri A., Mazzuoli-Weber G., Salmona M., Colnaghi L. (2022). Micro- and nanoplastics’ effects on protein folding and amyloidosis.. Int. J. Mol. Sci..

[18] Ragusa A., Svelato A., Santacroce C., Catalano P., Notarstefano V., Carnevali O., Papa F., Rongioletti M.C.A., Baiocco F., Draghi S., D’Amore E., Rinaldo D., Matta M., Giorgini E. (2021). Plasticenta: First evidence of microplastics in human placenta.. Environ. Int..

[19] Zhao Q., Zhu L., Weng J., Jin Z., Cao Y., Jiang H., Zhang Z. (2023). Detection and characterization of microplastics in the human testis and semen.. Sci. Total Environ..

[20] Chianese R., Coccurello R., Viggiano A., Scafuro M., Fiore M., Coppola G., Operto F.F., Fasano S., Laye S., Pierantoni R., Meccariello R. (2018). Impact of dietary fats on brain functions.. Curr. Neuropharmacol..

[21] Cryan J.F., O’Riordan K.J., Cowan C.S.M., Sandhu K.V., Bastiaanssen T.F.S., Boehme M., Codagnone M.G., Cussotto S., Fulling C., Golubeva A.V., Guzzetta K.E., Jaggar M., Long-Smith C.M., Lyte J.M., Martin J.A., Molinero-Perez A., Moloney G., Morelli E., Morillas E., O’Connor R., Cruz-Pereira J.S., Peterson V.L., Rea K., Ritz N.L., Sherwin E., Spichak S., Teichman E.M., van de Wouw M., Ventura-Silva A.P., Wallace-Fitzsimons S.E., Hyland N., Clarke G., Dinan T.G. (2019). The microbiota-gut-brain axis.. Physiol. Rev..

[22] DiSabato D.J., Quan N., Godbout J.P. (2016). Neuroinflammation: The devil is in the details.. J. Neurochem..

[23] Kwon H.S., Koh S.H. (2020). Neuroinflammation in neurodegenerative disorders: The roles of microglia and astrocytes.. Transl. Neurodegener..

[24] Meccariello R., Marino M., Mele E., Pastorino G.M.G., Operto F.F., Santoro A., Viggiano A. (2022). Neuroinflammation: Molecular mechanisms and therapeutic perspectives.. Cent. Nerv. Syst. Agents Med. Chem..

[25] Fried J.R. (2014). Polymer science and technology..

[26] Rujnić-Sokele M., Pilipović A. (2017). Challenges and opportunities of biodegradable plastics: A mini review.. Waste Manag. Res..

[27] Krueger M.C., Harms H., Schlosser D. (2015). Prospects for microbiological solutions to environmental pollution with plastics.. Appl. Microbiol. Biotechnol..

[28] Rahman M.H., Bhoi P.R. (2021). An overview of non-biodegradable bioplastics.. J. Clean. Prod..

[29] Lee W.T., van Muyden A., Bobbink F.D., Mensi M.D., Carullo J.R., Dyson P.J. (2022). Mechanistic classification and benchmarking of polyolefin depolymerization over silica-alumina-based catalysts.. Nat. Commun..

[30] Elgharbawy A.S., Ali R.M. (2022). A comprehensive review of the polyolefin composites and their properties.. Heliyon.

[31] Hees T., Zhong F., Stürzel M., Mülhaupt R. (2019). Tailoring hydrocarbon polymers and all‐hydrocarbon composites for circular economy.. Macromol. Rapid Commun..

[32] https://www.chemicalbook.com/.

[33] Yao Z., Seong H.J., Jang Y.S. (2022). Environmental toxicity and decomposition of polyethylene.. Ecotoxicol. Environ. Saf..

[34] Paxton N.C., Allenby M.C., Lewis P.M., Woodruff M.A. (2019). Biomedical applications of polyethylene.. Eur. Polym. J..

[35] Kumar L., Saha A. (2023). Khushbu, ; Warkar, S. G. Chapter 11 - Biodegradability of automotive plastics and composites.. Biodegradability of Conventional Plastics.

[36] Rani M. (2023). Biodegradability of Conventional Plastics..

[37] Li X., Meng L., Zhang Y., Qin Z., Meng L., Li C., Liu M. (2022). Research and application of polypropylene carbonate composite materials: A review.. Polymers.

[38] Blackley D.C. (1983). Plasticised polyvinyl chloride (PVC).. Synthetic Rubbers: Their Chemistry and Technology..

[39] Yu J., Sun L., Ma C., Qiao Y., Yao H. (2016). Thermal degradation of PVC: A review.. Waste Manag..

[40] Peng B.Y., Chen Z., Chen J., Yu H., Zhou X., Criddle C.S., Wu W.M., Zhang Y. (2020). Biodegradation of polyvinyl chloride (PVC) in tenebrio molitor (Coleoptera: Tenebrionidae) larvae.. Environ. Int..

[41] Lewandowski K., Skórczewska K. (2022). A brief review of poly(vinyl chloride) (PVC) recycling.. Polymers.

[42] Zhang Y., Pedersen J.N., Eser B.E., Guo Z. (2022). Biodegradation of polyethylene and polystyrene: From microbial deterioration to enzyme discovery.. Biotechnol. Adv..

[43] Kik K., Bukowska B., Sicińska P. (2020). Polystyrene nanoparticles: Sources, occurrence in the environment, distribution in tissues, accumulation and toxicity to various organisms.. Environ. Pollut..

[44] Pulido B.A., Habboub O.S., Aristizabal S.L., Szekely G., Nunes S.P. (2019). Recycled poly(ethylene terephthalate) for high temperature solvent resistant membranes.. ACS Appl. Polym. Mater..

[45] Hiraga K., Taniguchi I., Yoshida S., Kimura Y., Oda K. (2019). Biodegradation of waste PET.. EMBO Rep..

[46] Kushwaha A., Goswami L., Singhvi M., Kim B.S. (2023). Biodegradation of poly(ethylene terephthalate): Mechanistic insights, advances, and future innovative strategies.. Chem. Eng. J..

[47] Nisticò R. (2020). Polyethylene terephthalate (PET) in the packaging industry.. Polym. Test..

[48] Siracusa V., Blanco I. (2020). Bio-polyethylene (bio-pe), bio-polypropylene (bio-pp) and bio-poly(ethylene terephthalate) (bio-pet): Recent developments in bio-based polymers analogous to petroleum-derived ones for packaging and engineering applications.. Polymers.

[49] Wei B., Zhao Y., Wei Y., Yao J., Chen X., Shao Z. (2019). Morphology and properties of a new biodegradable material prepared from zein and poly(butylene adipate-terephthalate) by reactive blending.. ACS Omega.

[50] İlhan Z., Gümüşderelioğlu M. (2023). Oriented fibrous poly (butylene adipate-co-terephthalate) matrices with nanotopographic features: Production and characterization.. Colloids Surf. A Physicochem. Eng. Asp..

[51] Fu Y., Wu G., Bian X., Zeng J., Weng Y. (2020). Biodegradation behavior of poly(butylene adipate-co-terephthalate) (pbat), poly(lactic acid) (pla), and their blend in freshwater with sediment.. Molecules.

[52] Jia H., Zhang M., Weng Y., Zhao Y., Li C., Kanwal A. (2021). Degradation of poly(butylene adipate-co-terephthalate) by Stenotrophomonas sp. YCJ1 isolated from farmland soil.. J. Environ. Sci..

[53] Rafiqah S.A., Khalina A., Harmaen A.S., Tawakkal I.A., Zaman K., Asim M., Nurrazi M.N., Lee C.H. (2021). A review on properties and application of bio-based poly(butylene succinate).. Polymers.

[54] Boucher D.S. (2020). Solubility parameters and solvent affinities for polycaprolactone: A comparison of methods.. J. Appl. Polym. Sci..

[55] Heimowska A., Morawska M., Bocho-Janiszewska A. (2017). Biodegradation of poly(ε-caprolactone) in natural water environments.. Pol. J. Chem. Technol..

[56] Atanasova N., Paunova-Krasteva T., Stoitsova S., Radchenkova N., Boyadzhieva I., Petrov K., Kambourova M. (2021). Degradation of poly(ε-caprolactone) by a thermophilic community and brevibacillus thermoruber strain 7 isolated from bulgarian hot spring.. Biomolecules.

[57] Malikmammadov E., Tanir T.E., Kiziltay A., Hasirci V., Hasirci N. (2018). PCL and PCL-based materials in biomedical applications.. J. Biomater. Sci. Polym. Ed..

[58] Aliotta L., Seggiani M., Lazzeri A., Gigante V., Cinelli P. (2022). A brief review of poly (butylene succinate) (pbs) and its main copolymers: Synthesis, blends, composites, biodegradability, and applications.. Polymers.

[59] Cooper C.J., Mohanty A.K., Misra M. (2018). Electrospinning process and structure relationship of biobased poly(butylene succinate) for nanoporous fibers.. ACS Omega.

[60] Kim S.H., Cho J.Y., Cho D.H., Jung H.J., Kim B.C., Bhatia S.K., Park S.H., Park K., Yang Y.H. (2022). Acceleration of polybutylene succinate biodegradation by Terribacillus sp. JY49 isolated from a marine environment.. Polymers.

[61] Fredi G., Dorigato A. (2021). Recycling of bioplastic waste: A review.. Adv. Ind. Eng. Polym. Res..

[62] Casalini T., Rossi F., Castrovinci A., Perale G. (2019). A perspective on polylactic acid-based polymers use for nanoparticles synthesis and applications.. Front. Bioeng. Biotechnol..

[63] da Silva D., Kaduri M., Poley M., Adir O., Krinsky N., Shainsky-Roitman J., Schroeder A. (2018). Biocompatibility, biodegradation and excretion of polylactic acid (PLA) in medical implants and theranostic systems.. Chem. Eng. J..

[64] Bubpachat T., Sombatsompop N., Prapagdee B. (2018). Isolation and role of polylactic acid-degrading bacteria on degrading enzymes productions and PLA biodegradability at mesophilic conditions.. Polym. Degrad. Stabil..

[65] Balla E., Daniilidis V., Karlioti G., Kalamas T., Stefanidou M., Bikiaris N.D., Vlachopoulos A., Koumentakou I., Bikiaris D.N. (2021). Poly(lactic acid): A versatile biobased polymer for the future with multifunctional properties—from monomer synthesis, polymerization techniques and molecular weight increase to pla applications.. Polymers.

[66] https://www.chemsrc.com/en/cas/34346-01-5_1470921.html.

[67] Makadia H.K., Siegel S.J. (2011). Poly lactic-co-glycolic acid (plga) as biodegradable controlled drug delivery carrier.. Polymers.

[68] Kemme M., Prokesch I., Heinzel-Wieland R. (2011). Comparative study on the enzymatic degradation of poly(lactic-co-glycolic acid) by hydrolytic enzymes based on the colorimetric quantification of glycolic acid.. Polym. Test..

[69] Virlan M.J.R., Miricescu D., Totan A., Greabu M., Tanase C., Sabliov C.M., Caruntu C., Calenic B. (2015). Current uses of poly(lactic-co-glycolic acid) in the dental field: A comprehensive.. Rev. J. Chem..

[70] Keskin G., Kızıl G., Bechelany M., Pochat-Bohatier C., Öner M. (2017). Potential of polyhydroxyalkanoate (PHA) polymers family as substitutes of petroleum based polymers for packaging applications and solutions brought by their composites to form barrier materials.. Pure Appl. Chem..

[71] Vandi L.J., Chan C., Werker A., Richardson D., Laycock B., Pratt S. (2018). Wood-PHA composites: Mapping opportunities.. Polymers.

[72] Sehgal R., Gupta R. (2020). Polyhydroxyalkanoate and its efficient production: An eco-friendly approach towards development.. 3 Biotech.

[73] Volova T.G. (2015). Biodegradation of polyhydroxyalkanoates in natural soils.. J. Sib. Fed. Univ. Biol..

[74] Volova T.G., Prudnikova S.V., Vinogradova O.N., Syrvacheva D.A., Shishatskaya E.I. (2017). Microbial degradation of polyhydroxyalkanoates with different chemical compositions and their biodegradability.. Microb. Ecol..

[75] Koller M. (2018). Biodegradable and biocompatible polyhydroxy-alkanoates (pha): auspicious microbial macromolecules for pharmaceutical and therapeutic applications.. Molecules.

[76] Koster S., Bani-Estivals M., Bonuomo M., Bradley E., Chagnon M., Garcia M.L., Godts F., Gude T., Helling R., Paseiro-Losada P., Pieper G., Rennen M., Simat T., Spack L. (2016). Guidance on best practices on the risk assessment of non-intentionally added substances (NIAS) in food contact materials and articles.. ILSI Europe Report Series.

[77] Hahladakis J.N., Velis C.A., Weber R., Iacovidou E., Purnell P. (2018). An overview of chemical additives present in plastics: Migration, release, fate and environmental impact during their use, disposal and recycling.. J. Hazard. Mater..

[78] Tsochatzis E., Lopes J., Gika H., Theodoridis G. (2020). Polystyrene biodegradation by tenebrio molitor larvae: Identification of generated substances using a GC-MS untargeted screening method.. Polymers.

[79] Arianna P., Paola S., Luciano D.M., Loredana I. (2019). Non-listed nias exposure assessment: Comparison of different tools.. Chem. Eng. Trans..

[80] Geueke B. (2018). Fpf Dossier: Non-Intentionally Added Substances (Nias).

[81] (2015). Guidance on best available techniques and best environmental practices for the recycling and disposal of articles containing polybrominated diphenyl ethers (pbdes) listed under the stockholm convention on persistent organic pollutants.

[82] He Y.J., Qin Y., Zhang T.L., Zhu Y.Y., Wang Z.J., Zhou Z.S., Xie T.Z., Luo X.D. (2021). Migration of (non-) intentionally added substances and microplastics from microwavable plastic food containers.. J. Hazard. Mater..

[83] Muncke J., Andersson A-M., Backhaus T., Boucher J.M., Carney Almroth B., Castillo Castillo A., Chevrier J., Demeneix B.A., Emmanuel J.A., Fini J-B. (2020). Impacts of food contact chemicals on human health: A consensus statement.. Environ. Health.

[84] Santoro A., Chianese R., Troisi J., Richards S., Nori S.L., Fasano S., Guida M., Plunk E., Viggiano A., Pierantoni R., Meccariello R. (2019). Neuro-toxic and reproductive effects of BPA.. Curr. Neuropharmacol..

[85] Di Pietro P., D’Auria R., Viggiano A., Ciaglia E., Meccariello R., Russo R.D., Puca A.A., Vecchione C., Nori S.L., Santoro A. (2020). Bisphenol A induces DNA damage in cells exerting immune surveillance functions at peripheral and central level.. Chemosphere.

[86] Sree C.G., Buddolla V., Lakshmi B.A., Kim Y-J. (2023). Phthalate toxicity mechanisms: An update. Comp. Biochem. Physiol. Part C Toxicol.. Pharmacol..

[87] Yates M.R., Barlow C.Y. (2013). Life cycle assessments of biodegradable, commercial biopolymers—A critical review.. Resour. Conserv. Recycling.

[88] Porta R. (2019). The plastics sunset and the bio-plastics sunrise.. Coatings.

[89] Cao G., Cai Z. (2023). Getting health hazards of inhaled nano/] microplastics into focus: Expectations and challenges.. Environ. Sci. Technol..

[90] Nor N.H.M., Kooi M., Diepens N.J., Koelmans A.A. (2021). Lifetime accumulation of microplastic in children and adults.. Environ. Sci. Technol..

[91] Kole P.J., Löhr A.J., Van Belleghem F., Ragas A. (2017). Wear and tear of tyres: A stealthy source of microplastics in the environment.. Int. J. Environ. Res. Public Health.

[92] Prata J.C., da Costa J.P., Lopes I., Duarte A.C., Rocha-Santos T. (2020). Environmental exposure to microplastics: An overview on possible human health effects.. Sci. Total Environ..

[93] Grote K., Brüstle F., Vlacil A.K. (2023). Cellular and systemic effects of micro- and nanoplastics in mammals—what we know so far.. Materials.

[94] Karlsson H., Lindbom J., Ghafouri B., Lindahl M., Tagesson C., Gustafsson M., Ljungman A.G. (2011). Wear particles from studded tires and granite pavement induce pro-inflammatory alterations in human monocyte-derived macrophages: A proteomic study.. Chem. Res. Toxicol..

[95] Li Y., Shi T., Li X., Sun H., Xia X., Ji X., Zhang J., Liu M., Lin Y., Zhang R., Zheng Y., Tang J. (2022). Inhaled tire-wear microplastic particles induced pulmonary fibrotic injury *via* epithelial cytoskeleton rearrangement.. Environ. Int..

[96] Mantecca P., Sancini G., Moschini E., Farina F., Gualtieri M., Rohr A., Miserocchi G., Palestini P., Camatini M. (2009). Lung toxicity induced by intratracheal instillation of size-fractionated tire particles.. Toxicol. Lett..

[97] Islam S.U., Shehzad A., Ahmed M.B., Lee Y.S. (2020). Intranasal delivery of nanoformulations: A potential way of treatment for neurological disorders.. Molecules.

[98] Oberdörster G., Sharp Z., Atudorei V., Elder A., Gelein R., Kreyling W., Cox C. (2004). Translocation of inhaled ultrafine particles to the brain.. Inhal. Toxicol..

[99] Elder A., Gelein R., Silva V., Feikert T., Opanashuk L., Carter J., Potter R., Maynard A., Ito Y., Finkelstein J., Oberdörster G. (2006). Translocation of inhaled ultrafine manganese oxide particles to the central nervous system.. Environ. Health Perspect..

[100] Qi Y., Wei S., Xin T., Huang C., Pu Y., Ma J., Zhang C., Liu Y., Lynch I., Liu S. (2022). Passage of exogeneous fine particles from the lung into the brain in humans and animals.. Proc. Natl. Acad. Sci. USA.

[101] Chen G., Feng Q., Wang J. (2020). Mini-review of microplastics in the atmosphere and their risks to humans.. Sci. Total Environ..

[102] Karami A., Golieskardi A., Ho Y.B., Larat V., Salamatinia B. (2017). Microplastics in eviscerated flesh and excised organs of dried fish.. Sci. Rep..

[103] Sangkham S., Faikhaw O., Munkong N., Sakunkoo P., Arunlertaree C., Chavali M., Mousazadeh M., Tiwari A. (2022). A review on microplastics and nanoplastics in the environment: Their occurrence, exposure routes, toxic studies, and potential effects on human health.. Mar. Pollut. Bull..

[104] Güven O., Gökdağ K., Jovanović B., Kıdeyş A.E. (2017). Microplastic litter composition of the Turkish territorial waters of the mediterranean sea, and its occurrence in the gastrointestinal tract of fish.. Environ. Pollut..

[105] Han J., Yan J., Li K., Lin B., Lai W., Bian L., Jia R., Liu X., Xi Z. (2023). Distribution of micro-nano PS, DEHP, and/or MEHP in mice and nerve cell models *in vitro* after exposure to micro-nano PS and DEHP.. Toxics.

[106] Yang Z.S., Bai Y.L., Jin C.H., Na J., Zhang R., Gao Y., Pan G.W., Yan L.J., Sun W. (2022). Evidence on invasion of blood, adipose tissues, nervous system and reproductive system of mice after a single oral exposure: Nanoplastics versus microplastics.. Biomed. Environ. Sci..

[107] Lamparelli E.P., Marino M., Szychlinska M.A., Rocca N.D., Ciardulli M.C., Scala P., D’Auria R., Testa A., Viggiano A., Cappello F., Meccariello R., Porta G.D., Santoro A. (2023). The other side of plastics: Bioplastic-based nanoparticles for drug delivery systems in the brain.. Pharmaceutics.

[108] Lee J.A., Kim M.K., Paek H.J., Kim Y.R., Kim M.K., Lee J.K., Jeong J., Choi S.J., Choi S-J. (2014). Tissue distribution and excretion kinetics of orally administered silica nanoparticles in rats.. Int. J. Nanomedicine.

[109] Khan A.W., Farooq M., Hwang M.J., Haseeb M., Choi S. (2023). Autoimmune neuroinflammatory diseases: Role of interleukins.. Int. J. Mol. Sci..

[110] Sofroniew M.V. (2009). Molecular dissection of reactive astrogliosis and glial scar formation.. Trends Neurosci..

[111] Takata F., Nakagawa S., Matsumoto J., Dohgu S. (2021). Blood-brain barrier dysfunction amplifies the development of neuroinflammation: Understanding of cellular events in brain microvascular endothelial cells for prevention and treatment of BBB dysfunction.. Front. Cell. Neurosci..

[112] Takeshita Y., Obermeier B., Cotleur A.C., Spampinato S.F., Shimizu F., Yamamoto E., Sano Y., Kryzer T.J., Lennon V.A., Kanda T., Ransohoff R.M. (2017). Effects of neuromyelitis optica–IgG at the blood-brain barrier *in vitro*.. Neurol. Neuroimmunol. Neuroinflamm..

[113] Linnerbauer M., Rothhammer V. (2020). Protective functions of reactive astrocytes following central nervous system insult.. Front. Immunol..

[114] Rostami J., Fotaki G., Sirois J., Mzezewa R., Bergström J., Essand M., Healy L., Erlandsson A. (2020). Astrocytes have the capacity to act as antigen-presenting cells in the Parkinson’s disease brain.. J. Neuroinflammation.

[115] Ranaivo H.R., Hodge J.N., Choi N., Wainwright M.S. (2012). Albumin induces upregulation of matrix metalloproteinase-9 in astrocytes *via* MAPK and reactive oxygen species-dependent pathways.. J. Neuroinflammation.

[116] Corrigan F., Mander K.A., Leonard A.V., Vink R. (2016). Neurogenic inflammation after traumatic brain injury and its potentiation of classical inflammation.. J. Neuroinflammation.

[117] Sulimai N., Lominadze D. (2020). Fibrinogen and neuroinflammation during traumatic brain injury.. Mol. Neurobiol..

[118] Katsouri L., Birch A.M., Renziehausen A.W.J., Zach C., Aman Y., Steeds H., Bonsu A., Palmer E.O.C., Mirzaei N., Ries M., Sastre M. (2020). Ablation of reactive astrocytes exacerbates disease pathology in a model of Alzheimer’s disease.. Glia.

[119] Colombo E., Farina C. (2016). Astrocytes: Key regulators of neuroinflammation.. Trends Immunol..

[120] Salman M.M., Kitchen P., Halsey A., Wang M.X., Törnroth-Horsefield S., Conner A.C., Badaut J., Iliff J.J., Bill R.M. (2022). Emerging roles for dynamic aquaporin-4 subcellular relocalization in CNS water homeostasis.. Brain.

[121] Iliff J.J., Wang M., Liao Y., Plogg B.A., Peng W., Gundersen G.A., Benveniste H., Vates G.E., Deane R., Goldman S.A., Nagelhus E.A., Nedergaard M. (2012). A paravascular pathway facilitates CSF flow through the brain parenchyma and the clearance of interstitial solutes, including amyloid β.. Sci. Transl. Med..

[122] Aspelund A., Antila S., Proulx S.T., Karlsen T.V., Karaman S., Detmar M., Wiig H., Alitalo K. (2015). A dural lymphatic vascular system that drains brain interstitial fluid and macromolecules.. J. Exp. Med..

[123] Louveau A., Smirnov I., Keyes T.J., Eccles J.D., Rouhani S.J., Peske J.D., Derecki N.C., Castle D., Mandell J.W., Lee K.S., Harris T.H., Kipnis J. (2015). Structural and functional features of central nervous system lymphatic vessels.. Nature.

[124] Mogensen F.L.H., Delle C., Nedergaard M. (2021). The glymphatic system (En)during inflammation.. Int. J. Mol. Sci..

[125] Louveau A., Herz J., Alme M.N., Salvador A.F., Dong M.Q., Viar K.E., Herod S.G., Knopp J., Setliff J.C., Lupi A.L., Da Mesquita S., Frost E.L., Gaultier A., Harris T.H., Cao R., Hu S., Lukens J.R., Smirnov I., Overall C.C., Oliver G., Kipnis J. (2018). CNS lymphatic drainage and neuroinflammation are regulated by meningeal lymphatic vasculature.. Nat. Neurosci..

[126] Hsu S.J., Zhang C., Jeong J., Lee S., McConnell M., Utsumi T., Iwakiri Y. (2021). Enhanced meningeal lymphatic drainage ameliorates neuroinflammation and hepatic encephalopathy in cirrhotic rats.. Gastroenterology.

[127] Da Mesquita S., Papadopoulos Z., Dykstra T., Brase L., Farias F.G., Wall M., Jiang H., Kodira C.D., de Lima K.A., Herz J., Louveau A., Goldman D.H., Salvador A.F., Onengut-Gumuscu S., Farber E., Dabhi N., Kennedy T., Milam M.G., Baker W., Smirnov I., Rich S.S., Benitez B.A., Karch C.M., Perrin R.J., Farlow M., Chhatwal J.P., Holtzman D.M., Cruchaga C., Harari O., Kipnis J. (2021). Meningeal lymphatics affect microglia responses and anti-Aβ immunotherapy.. Nature.

[128] Hamby M.E., Coppola G., Ao Y., Geschwind D.H., Khakh B.S., Sofroniew M.V. (2012). Inflammatory mediators alter the astrocyte transcriptome and calcium signaling elicited by multiple G-protein-coupled receptors.. J. Neurosci..

[129] Liddelow S.A., Guttenplan K.A., Clarke L.E., Bennett F.C., Bohlen C.J., Schirmer L., Bennett M.L., Münch A.E., Chung W.S., Peterson T.C., Wilton D.K., Frouin A., Napier B.A., Panicker N., Kumar M., Buckwalter M.S., Rowitch D.H., Dawson V.L., Dawson T.M., Stevens B., Barres B.A. (2017). Neurotoxic reactive astrocytes are induced by activated microglia.. Nature.

[130] Baxter P.S., Dando O., Emelianova K., He X., McKay S., Hardingham G.E., Qiu J. (2021). Microglial identity and inflammatory responses are controlled by the combined effects of neurons and astrocytes.. Cell Rep..

[131] Santoro A., Spinelli C.C., Martucciello S., Nori S.L., Capunzo M., Puca A.A., Ciaglia E. (2018). Innate immunity and cellular senescence: The good and the bad in the developmental and aged brain.. J. Leukoc. Biol..

[132] Lima M.N., Barbosa-Silva M.C., Maron-Gutierrez T. (2022). Microglial priming in infections and its risk to neurodegenerative diseases.. Front. Cell. Neurosci..

[133] Borst K., Dumas A.A., Prinz M. (2021). Microglia: Immune and non-immune functions.. Immunity.

[134] Rutsch A., Kantsjö J.B., Ronchi F. (2020). The gut-brain axis: How microbiota and host inflammasome influence brain physiology and pathology.. Front. Immunol..

[135] Pokusaeva K., Johnson C., Luk B., Uribe G., Fu Y., Oezguen N., Matsunami R.K., Lugo M., Major A., Mori-Akiyama Y., Hollister E.B., Dann S.M., Shi X.Z., Engler D.A., Savidge T., Versalovic J. (2017). GABA ‐producing Bifidobacterium dentium modulates visceral sensitivity in the intestine.. Neurogastroenterol. Motil..

[136] Roth W., Zadeh K., Vekariya R., Ge Y., Mohamadzadeh M. (2021). Tryptophan metabolism and gut-brain homeostasis.. Int. J. Mol. Sci..

[137] Glebov K., Löchner M., Jabs R., Lau T., Merkel O., Schloss P., Steinhäuser C., Walter J. (2015). Serotonin stimulates secretion of exosomes from microglia cells.. Glia.

[138] Rothhammer V., Borucki D.M., Tjon E.C., Takenaka M.C., Chao C.C., Ardura-Fabregat A., de Lima K.A., Gutiérrez-Vázquez C., Hewson P., Staszewski O., Blain M., Healy L., Neziraj T., Borio M., Wheeler M., Dragin L.L., Laplaud D.A., Antel J., Alvarez J.I., Prinz M., Quintana F.J. (2018). Microglial control of astrocytes in response to microbial metabolites.. Nature.

[139] Smith S.E.P., Li J., Garbett K., Mirnics K., Patterson P.H. (2007). Maternal immune activation alters fetal brain development through interleukin-6.. J. Neurosci..

[140] Choi G.B., Yim Y.S., Wong H., Kim S., Kim H., Kim S.V., Hoeffer C.A., Littman D.R., Huh J.R. (2016). The maternal interleukin-17a pathway in mice promotes autism-like phenotypes in offspring.. Science.

[141] Qu X., Yu X., Liu J., Wang J., Liu J. (2016). Pro-inflammatory cytokines are elevated in pregnant women with systemic lupus erythematosus in association with the activation of TLR4.. Clin. Lab..

[142] Vijay K. (2018). Toll-like receptors in immunity and inflammatory diseases: Past, present, and future.. Int. Immunopharmacol..

[143] Mattei D., Ivanov A., Ferrai C., Jordan P., Guneykaya D., Buonfiglioli A., Schaafsma W., Przanowski P., Deuther-Conrad W., Brust P., Hesse S., Patt M., Sabri O., Ross T.L., Eggen B.J.L., Boddeke E.W.G.M., Kaminska B., Beule D., Pombo A., Kettenmann H., Wolf S.A. (2017). Maternal immune activation results in complex microglial transcriptome signature in the adult offspring that is reversed by minocycline treatment.. Transl. Psychiatry.

[144] Matcovitch-Natan O., Winter D.R., Giladi A., Vargas Aguilar S., Spinrad A., Sarrazin S., Ben-Yehuda H., David E., Zelada González F., Perrin P., Keren-Shaul H., Gury M., Lara-Astaiso D., Thaiss C.A., Cohen M., Bahar Halpern K., Baruch K., Deczkowska A., Lorenzo-Vivas E., Itzkovitz S., Elinav E., Sieweke M.H., Schwartz M., Amit I. (2016). Microglia development follows a stepwise program to regulate brain homeostasis.. Science.

[145] de Souza D.F., Wartchow K.M., Lunardi P.S., Brolese G., Tortorelli L.S., Batassini C., Biasibetti R., Gonçalves C.A. (2015). Changes in astroglial markers in a maternal immune activation model of schizophrenia in wistar rats are dependent on sex.. Front. Cell. Neurosci..

[146] McCarthy M.M., Wright C.L. (2017). Convergence of sex differences and the neuroimmune system in autism spectrum disorder.. Biol. Psychiatry.

[147] Vilella A.J., Severin J., Ureta-Vidal A., Heng L., Durbin R., Birney E. (2009). Ensemblcompara genetrees: Complete, duplication-aware phylogenetic trees in vertebrates.. Genome Res..

[148] Golzio C., Willer J., Talkowski M.E., Oh E.C., Taniguchi Y., Jacquemont S., Reymond A., Sun M., Sawa A., Gusella J.F., Kamiya A., Beckmann J.S., Katsanis N. (2012). KCTD13 is a major driver of mirrored neuroanatomical phenotypes of the 16p11.2 copy number variant.. Nature.

[149] Guo S. (2004). Linking genes to brain, behavior and neurological diseases: What can we learn from zebrafish?. Genes Brain Behav..

[150] Schmidt R., Strähle U., Scholpp S. (2013). Neurogenesis in zebrafish – from embryo to adult.. Neural Dev..

[151] Wullimann M.F., Mueller T. (2004). Teleostean and mammalian forebrains contrasted: Evidence from genes to behavior.. J. Comp. Neurol..

[152] Mhalhel K., Sicari M., Pansera L., Chen J., Levanti M., Diotel N., Rastegar S., Germanà A., Montalbano G. (2023). Zebrafish: A model deciphering the impact of flavonoids on neurodegenerative disorders.. Cells.

[153] Cosacak M.I., Bhattarai P., De Jager P.L., Menon V., Tosto G., Kizil C. (2022). Single cell/nucleus transcriptomics comparison in zebrafish and humans reveals common and distinct molecular responses to alzheimer’s disease.. Cells.

[154] Bhattarai P., Thomas A.K., Cosacak M.I., Papadimitriou C., Mashkaryan V., Froc C., Reinhardt S., Kurth T., Dahl A., Zhang Y., Kizil C. (2016). IL4/STAT6 signaling activates neural stem cell proliferation and neurogenesis upon Amyloid-β42 aggregation in adult zebrafish brain.. Cell Rep..

[155] Botterell Z.L.R., Beaumont N., Dorrington T., Steinke M., Thompson R.C., Lindeque P.K. (2019). Bioavailability and effects of microplastics on marine zooplankton: A review.. Environ. Pollut..

[156] Prata J.C., da Costa J.P., Lopes I., Duarte A.C., Rocha-Santos T. (2019). Effects of microplastics on microalgae populations: A critical review.. Sci. Total Environ..

[157] Vo H.C., Pham M.H. (2021). Ecotoxicological effects of microplastics on aquatic organisms: A review.. Environ. Sci. Pollut. Res. Int..

[158] Prüst M., Meijer J., Westerink R.H.S. (2020). The plastic brain: Neurotoxicity of micro- and nanoplastics.. Part. Fibre Toxicol..

[159] Sarasamma S., Audira G., Siregar P., Malhotra N., Lai Y.H., Liang S.T., Chen J.R., Chen K.H.C., Hsiao C.D. (2020). Nanoplastics cause neurobehavioral impairments, reproductive and oxidative damages, and biomarker responses in zebrafish: Throwing up alarms of wide spread health risk of exposure.. Int. J. Mol. Sci..

[160] Xiang C., Chen H., Liu X., Dang Y., Li X., Yu Y., Li B., Li X., Sun Y., Ding P., Hu G. (2023). UV-aged microplastics induces neurotoxicity by affecting the neurotransmission in larval zebrafish.. Chemosphere.

[161] Yu H., Chen Q., Qiu W., Ma C., Gao Z., Chu W., Shi H. (2022). Concurrent water- and foodborne exposure to microplastics leads to differential microplastic ingestion and neurotoxic effects in zebrafish.. Water Res..

[162] Lee H., Tran C.M., Jeong S., Kim S.S., Bae M.A., Kim K-T. (2022). Seizurogenic effect of perfluorooctane sulfonate in zebrafish larvae.. Neurotoxicology.

[163] Ding P., Xiang C., Li X., Chen H., Shi X., Li X., Huang C., Yu Y., Qi J., Li A.J., Zhang L., Hu G. (2023). Photoaged microplastics induce neurotoxicity *via* oxidative stress and abnormal neurotransmission in zebrafish larvae (Danio rerio).. Sci. Total Environ..

[164] Umamaheswari S., Priyadarshinee S., Bhattacharjee M., Kadirvelu K., Ramesh M. (2021). Exposure to polystyrene microplastics induced gene modulated biological responses in zebrafish (Danio rerio).. Chemosphere.

[165] Teng M., Zhao X., Wu F., Wang C., Wang C., White J.C., Zhao W., Zhou L., Yan S., Tian S. (2022). Charge-specific adverse effects of polystyrene nanoplastics on zebrafish (Danio rerio) development and behavior.. Environ. Int..

[166] Mattsson K., Johnson E.V., Malmendal A., Linse S., Hansson L.A., Cedervall T. (2017). Brain damage and behavioural disorders in fish induced by plastic nanoparticles delivered through the food chain.. Sci. Rep..

[167] Barboza L.G.A., Otero X.L., Fernández E.V., Vieira L.R., Fernandes J.O., Cunha S.C., Guilhermino L. (2023). Are microplastics contributing to pollution-induced neurotoxicity? A pilot study with wild fish in a real scenario.. Heliyon.

[168] Ding J., Zhang S., Razanajatovo R.M., Zou H., Zhu W. (2018). Accumulation, tissue distribution, and biochemical effects of polystyrene microplastics in the freshwater fish red tilapia (Oreochromis niloticus).. Environ. Pollut..

[169] Xiong F., Liu J., Xu K., Huang J., Wang D., Li F., Wang S., Zhang J., Pu Y., Sun R. (2023). Microplastics induce neurotoxicity in aquatic animals at environmentally realistic concentrations: A meta-analysis.. Environ. Pollut..

[170] Lionetto M.G., Caricato R., Calisi A., Giordano M.E., Schettino T. (2013). Acetylcholinesterase as a biomarker in environmental and occupational medicine: New insights and future perspectives.. BioMed Res. Int..

[171] Morais L.H., Schreiber H.L., Mazmanian S.K. (2021). The gut microbiota–brain axis in behaviour and brain disorders.. Nat. Rev. Microbiol..

[172] Qiao R., Sheng C., Lu Y., Zhang Y., Ren H., Lemos B. (2019). Microplastics induce intestinal inflammation, oxidative stress, and disorders of metabolome and microbiome in zebrafish.. Sci. Total Environ..

[173] Zhao Y., Qin Z., Huang Z., Bao Z., Luo T., Jin Y. (2021). Effects of polyethylene microplastics on the microbiome and metabolism in larval zebrafish.. Environ. Pollut..

[174] Teng M., Zhao X., Wang C., Wang C., White J.C., Zhao W., Zhou L., Duan M., Wu F. (2022). Polystyrene nanoplastics toxicity to zebrafish: Dysregulation of the brain–intestine–microbiota axis.. ACS Nano.

[175] Luan J., Zhang S., Xu Y., Wen L., Feng X. (2023). Effects of microplastic exposure on the early developmental period and circadian rhythm of zebrafish (Danio rerio): A comparative study of polylactic acid and polyglycolic acid.. Ecotoxicol. Environ. Saf..

[176] Chagas T.Q., Freitas Í.N., Montalvão M.F., Nobrega R.H., Machado M.R.F., Charlie-Silva I., Araújo A.P.C., Guimarães A.T.B., Alvarez T.G.S., Malafaia G. (2021). Multiple endpoints of polylactic acid biomicroplastic toxicity in adult zebrafish (Danio rerio).. Chemosphere.

[177] de Oliveira J.P.J., Estrela F.N., Rodrigues A.S.L., Guimarães A.T.B., Rocha T.L., Malafaia G. (2021). Behavioral and biochemical consequences of Danio rerio larvae exposure to polylactic acid bioplastic.. J. Hazard. Mater..

[178] Duan Z., Cheng H., Duan X., Zhang H., Wang Y., Gong Z., Zhang H., Sun H., Wang L. (2022). Diet preference of zebrafish (Danio rerio) for bio-based polylactic acid microplastics and induced intestinal damage and microbiota dysbiosis.. J. Hazard. Mater..

[179] Zhang X., Xia M., Su X., Yuan P., Li X., Zhou C., Wan Z., Zou W. (2021). Photolytic degradation elevated the toxicity of polylactic acid microplastics to developing zebrafish by triggering mitochondrial dysfunction and apoptosis.. J. Hazard. Mater..

[180] Chen Q., Gundlach M., Yang S., Jiang J., Velki M., Yin D., Hollert H. (2017). Quantitative investigation of the mechanisms of microplastics and nanoplastics toward zebrafish larvae locomotor activity.. Sci. Total Environ..

[181] Wan Z., Wang C., Zhou J., Shen M., Wang X., Fu Z., Jin Y. (2019). Effects of polystyrene microplastics on the composition of the microbiome and metabolism in larval zebrafish.. Chemosphere.

[182] Mak C.W., Ching-Fong Yeung K., Chan K.M. (2019). Acute toxic effects of polyethylene microplastic on adult zebrafish.. Ecotoxicol. Environ. Saf..

[183] Santos D., Félix L., Luzio A., Parra S., Cabecinha E., Bellas J., Monteiro S.M. (2020). Toxicological effects induced on early life stages of zebrafish (Danio rerio) after an acute exposure to microplastics alone or co-exposed with copper.. Chemosphere.

[184] Santos D., Félix L., Luzio A., Parra S., Bellas J., Monteiro S.M. (2021). Single and combined acute and subchronic toxic effects of microplastics and copper in zebrafish (Danio rerio) early life stages.. Chemosphere.

[185] Xue Y.H., Feng L.S., Xu Z.Y., Zhao F.Y., Wen X.L., Jin T., Sun Z.X. (2021). The time-dependent variations of zebrafish intestine and gill after polyethylene microplastics exposure.. Ecotoxicology.

[186] Limonta G., Mancia A., Abelli L., Fossi M.C., Caliani I., Panti C. (2021). Effects of microplastics on head kidney gene expression and enzymatic biomarkers in adult zebrafish.. Comp. Biochem. Physiol. C Toxicol. Pharmacol..

[187] Guimarães A.T.B., Charlie-Silva I., Malafaia G. (2021). Toxic effects of naturally-aged microplastics on zebrafish juveniles: A more realistic approach to plastic pollution in freshwater ecosystems.. J. Hazard. Mater..

[188] Sheng C., Zhang S., Zhang Y. (2021). The influence of different polymer types of microplastics on adsorption, accumulation, and toxicity of triclosan in zebrafish.. J. Hazard. Mater..

[189] Bhagat J., Zang L., Nakayama H., Nishimura N., Shimada Y. (2021). Effects of nanoplastic on toxicity of azole fungicides (ketoconazole and fluconazole) in zebrafish embryos.. Sci. Total Environ..

[190] Zhu J., Zhang Y., Xu Y., Wang L., Wu Q., Zhang Z., Li L. (2022). Effects of microplastics on the accumulation and neurotoxicity of methylmercury in zebrafish larvae.. Mar. Environ. Res..

[191] Liu Y., Wang Y., Li N., Jiang S. (2022). Avobenzone and nanoplastics affect the development of zebrafish nervous system and retinal system and inhibit their locomotor behavior.. Sci. Total Environ..

[192] Santos D., Luzio A., Bellas J., Monteiro S.M. (2022). Microplastics- and copper-induced changes in neurogenesis and DNA methyltransferases in the early life stages of zebrafish.. Chem. Biol. Interact..

[193] Santos D., Luzio A., Félix L., Bellas J., Monteiro S.M. (2022). Oxidative stress, apoptosis and serotonergic system changes in zebrafish (Danio rerio) gills after long-term exposure to microplastics and copper.. Comp. Biochem. Physiol. C Toxicol. Pharmacol..

[194] Jeong S., Jang S., Kim S.S., Bae M.A., Shin J., Lee K.B., Kim K.T. (2022). Size-dependent seizurogenic effect of polystyrene microplastics in zebrafish embryos.. J. Hazard. Mater..

[195] Hanslik L., Huppertsberg S., Kämmer N., Knepper T.P., Braunbeck T. (2022). Rethinking the relevance of microplastics as vector for anthropogenic contaminants: Adsorption of toxicants to microplastics during exposure in a highly polluted stream - Analytical quantification and assessment of toxic effects in zebrafish (Danio rerio).. Sci. Total Environ..

[196] Aliakbarzadeh F., Rafiee M., Khodagholi F., Khorramizadeh M.R., Manouchehri H., Eslami A., Sayehmiri F., Mohseni-Bandpei A. (2023). Adverse effects of polystyrene nanoplastic and its binary mixtures with nonylphenol on zebrafish nervous system: From oxidative stress to impaired neurotransmitter system.. Environ. Pollut..

[197] Zhang C., Li Y., Yu H., Ye L., Li T., Zhang X., Wang C., Li P., Ji H., Gao Q., Dong S. (2023). Nanoplastics promote arsenic-induced ROS accumulation, mitochondrial damage and disturbances in neurotransmitter metabolism of zebrafish (Danio rerio).. Sci. Total Environ..

[198] Martin-Folgar R., Torres-Ruiz M., de Alba M., Cañas-Portilla A.I., González M.C., Morales M. (2023). Molecular effects of polystyrene nanoplastics toxicity in zebrafish embryos (Danio rerio).. Chemosphere.

[199] Zhou R., Zhou D., Yang S., Shi Z., Pan H., Jin Q., Ding Z. (2023). Neurotoxicity of polystyrene nanoplastics with different particle sizes at environment-related concentrations on early zebrafish embryos.. Sci. Total Environ..

[200] Torres-Ruiz M., de Alba González M., Morales M., Martin-Folgar R., González M.C., Cañas-Portilla A.I., De la Vieja A. (2023). Neurotoxicity and endocrine disruption caused by polystyrene nanoparticles in zebrafish embryo.. Sci. Total Environ..

[201] Wang Q., Chen G., Tian L., Kong C., Gao D., Chen Y., Junaid M., Wang J. (2023). Neuro- and hepato-toxicity of polystyrene nanoplastics and polybrominated diphenyl ethers on early life stages of zebrafish.. Sci. Total Environ..

[202] Murali K., Kenesei K., Li Y., Demeter K., Környei Z., Madarász E. (2015). Uptake and bio-reactivity of polystyrene nanoparticles is affected by surface modifications, ageing and LPS adsorption: *In vitro* studies on neural tissue cells.. Nanoscale.

[203] Schirinzi G.F., Pérez-Pomeda I., Sanchís J., Rossini C., Farré M., Barceló D. (2017). Cytotoxic effects of commonly used nanomaterials and microplastics on cerebral and epithelial human cells.. Environ. Res..

[204] Hoelting L., Scheinhardt B., Bondarenko O., Schildknecht S., Kapitza M., Tanavde V., Tan B., Lee Q.Y., Mecking S., Leist M., Kadereit S. (2013). A 3-dimensional human embryonic stem cell (hESC)-derived model to detect developmental neurotoxicity of nanoparticles.. Arch. Toxicol..

[205] Shan S., Zhang Y., Zhao H., Zeng T., Zhao X. (2022). Polystyrene nanoplastics penetrate across the blood-brain barrier and induce activation of microglia in the brain of mice.. Chemosphere.

[206] Sun J., Wang Y., Du Y., Zhang W., Liu Z., Bai J., Cui G., Du Z. (2023). Involvement of the JNK/HO 1/FTH1 signaling pathway in nanoplastic induced inflammation and ferroptosis of BV2 microglia cells.. Int. J. Mol. Med..

[207] Kwon W., Kim D., Kim H.Y., Jeong S.W., Lee S.G., Kim H.C., Lee Y.J., Kwon M.K., Hwang J.S., Han J.E., Park J.K., Lee S.J., Choi S.K. (2022). Microglial phagocytosis of polystyrene microplastics results in immune alteration and apoptosis *in vitro* and *in vivo*.. Sci. Total Environ..

[208] Ban M., Shimoda R., Chen J. (2021). Investigation of nanoplastic cytotoxicity using SH-SY5Y human neuroblastoma cells and polystyrene nanoparticles.. Toxicol. In Vitro.

[209] Nie J., Shen Y., Roshdy M., Cheng X., Wang G., Yang X. (2021). Polystyrene nanoplastics exposure caused defective neural tube morphogenesis through caveolae-mediated endocytosis and faulty apoptosis.. Nanotoxicology.

[210] Tang Q., Li T., Chen K., Deng X., Zhang Q., Tang H., Shi Z., Zhu T., Zhu J. (2022). PS-NPs induced neurotoxic effects in shsy-5y cells *via* autophagy activation and mitochondrial dysfunction.. Brain Sci..

[211] Hua T., Kiran S., Li Y., Sang Q.X.A. (2022). Microplastics exposure affects neural development of human pluripotent stem cell-derived cortical spheroids.. J. Hazard. Mater..

[212] Jeong J.H., Kang S.H., Kim J.H., Yu K.S., Lee I.H., Lee Y.J., Lee J.H., Lee N.S., Jeong Y.G., Kim D.K., Kim G.H., Lee S.H., Hong S.K., Han S.Y., Kang B.S. (2014). Protective effects of poly(lactic-co-glycolic acid) nanoparticles loaded with erythropoietin stabilized by sodium cholate against glutamate-induced neurotoxicity.. J. Nanosci. Nanotechnol..

[213] Jin H., Yang C., Jiang C., Li L., Pan M., Li D., Han X., Ding J. (2022). Evaluation of neurotoxicity in BALB/c mice following chronic exposure to polystyrene microplastics.. Environ. Health Perspect..

[214] Lee C.W., Hsu L.F., Wu I.L., Wang Y.L., Chen W.C., Liu Y.J., Yang L.T., Tan C.L., Luo Y.H., Wang C.C., Chiu H.W., Yang T.C.K., Lin Y.Y., Chang H.A., Chiang Y.C., Chen C.H., Lee M.H., Peng K.T., Huang C.C.Y. (2022). Exposure to polystyrene microplastics impairs hippocampus-dependent learning and memory in mice.. J. Hazard. Mater..

[215] Zaheer J., Kim H., Ko I.O., Jo E.K., Choi E.J., Lee H.J., Shim I., Woo H., Choi J., Kim G.H., Kim J.S. (2022). Pre/post-natal exposure to microplastic as a potential risk factor for autism spectrum disorder.. Environ. Int..

[216] Sincihu Y., Lusno M.F.D., Mulyasari T.M., Elias S.M., Sudiana I.K., Kusumastuti K., Sulistyorini L., Keman S. (2023). Wistar rats hippocampal neurons response to blood low-density polyethylene microplastics: A pathway analysis of SOD, CAT, MDA, 8-OHdG expression in hippocampal neurons and blood serum Aβ42 levels.. Neuropsychiatr. Dis. Treat..

[217] Supraja P., Tripathy S., Singh R., Singh V., Chaudhury G., Singh S.G. (2021). Towards point-of-care diagnosis of alzheimer’s disease: Multi-analyte based portable chemiresistive platform for simultaneous detection of β-amyloid (1-40) and (1-42) in plasma.. Biosens. Bioelectron..

[218] Yang D., Zhu J., Zhou X., Pan D., Nan S., Yin R., Lei Q., Ma N., Zhu H., Chen J., Han L., Ding M., Ding Y. (2022). Polystyrene micro- and nano-particle coexposure injures fetal thalamus by inducing ROS-mediated cell apoptosis.. Environ. Int..

[219] McConnell E.R., McClain M.A., Ross J., LeFew W.R., Shafer T.J. (2012). Evaluation of multi-well microelectrode arrays for neurotoxicity screening using a chemical training set.. Neurotoxicology.

[220] Hu M., Palić D. (2020). Micro- and nano-plastics activation of oxidative and inflammatory adverse outcome pathways.. Redox Biol..

[221] Prokić M.D., Radovanović T.B., Gavrić J.P., Faggio C. (2019). Ecotoxicological effects of microplastics: Examination of biomarkers, current state and future perspectives.. Trends Analyt. Chem..

[222] Zheng J., Suh S. (2019). Strategies to reduce the global carbon footprint of plastics.. Nat. Clim. Chang..

[223] Landrigan P.J., Stegeman J.J., Fleming L.E., Allemand D., Anderson D.M., Backer L.C., Brucker-Davis F., Chevalier N., Corra L., Czerucka D., Bottein M.Y.D., Demeneix B., Depledge M., Deheyn D.D., Dorman C.J., Fénichel P., Fisher S., Gaill F., Galgani F., Gaze W.H., Giuliano L., Grandjean P., Hahn M.E., Hamdoun A., Hess P., Judson B., Laborde A., McGlade J., Mu J., Mustapha A., Neira M., Noble R.T., Pedrotti M.L., Reddy C., Rocklöv J., Scharler U.M., Shanmugam H., Taghian G., Van de Water J.A.J.M., Vezzulli L., Weihe P., Zeka A., Raps H., Rampal P. (2020). Human health and ocean pollution.. Ann. Glob. Health.

[224] Gore A.C., Chappell V.A., Fenton S.E., Flaws J.A., Nadal A., Prins G.S., Toppari J., Zoeller R.T. (2015). EDC-2: The endocrine society’s second scientific statement on endocrine-disrupting chemicals.. Endocr. Rev..

[225] Woskie S.R., Bello A., Rennix C., Jiang L., Trivedi A.N., Savitz D.A. (2023). Burn pit exposure assessment to support a cohort study of US veterans of the wars in Iraq and Afghanistan.. J. Occup. Environ. Med..

[226] Re D.B., Yan B., Calderón-Garcidueñas L., Andrew A.S., Tischbein M., Stommel E.W. (2022). A perspective on persistent toxicants in veterans and amyotrophic lateral sclerosis: Identifying exposures determining higher ALS risk.. J. Neurol..

[227] Du Preez M., Van der Merwe D., Wyma L., Ellis S.M. (2021). Assessing knowledge and use practices of plastic food packaging among young adults in South Africa: Concerns about chemicals and health.. Int. J. Environ. Res. Public Health.

[228] Landrigan P.J., Raps H., Cropper M., Bald C., Brunner M., Canonizado E.M., Charles D., Chiles T.C., Donohue M.J., Enck J., Fenichel P., Fleming L.E., Ferrier-Pages C., Fordham R., Gozt A., Griffin C., Hahn M.E., Haryanto B., Hixson R., Ianelli H., James B.D., Kumar P., Laborde A., Law K.L., Martin K., Mu J., Mulders Y., Mustapha A., Niu J., Pahl S., Park Y., Pedrotti M.L., Pitt J.A., Ruchirawat M., Seewoo B.J., Spring M., Stegeman J.J., Suk W., Symeonides C., Takada H., Thompson R.C., Vicini A., Wang Z., Whitman E., Wirth D., Wolff M., Yousuf A.K., Dunlop S. (2023). The minderoo-monaco commission on plastics and human health.. Ann. Glob. Health.

[229] Pinilla L., Aguilar E., Dieguez C., Millar R.P., Tena-Sempere M. (2012). Kisspeptins and reproduction: Physiological roles and regulatory mechanisms.. Physiol. Rev..

[230] Pierantoni R., Cobellis G., Meccariello R., Fasano S. (2002). Evolutionary aspects of cellular communication in the vertebrate hypothalamo–hypophysio–gonadal axis.. International Review of Cytology..

[231] Wang J., Li Y., Lu L., Zheng M., Zhang X., Tian H., Wang W., Ru S. (2019). Polystyrene microplastics cause tissue damages, sexspecific reproductive disruption and transgenerational effects in marine medaka (*Oryzias melastigma*).. Environ. Pollut..

[232] Zhu M., Chernick M., Rittschof D., Hinton D.E. (2020). Chronic dietary exposure to polystyrene microplastics in maturing Japanese medaka (Oryzias latipes).. Aquat. Toxicol..

[233] Sussarellu R., Suquet M., Thomas Y., Lambert C., Fabioux C., Pernet M.E.J., Le Goïc N., Quillien V., Mingant C., Epelboin Y., Corporeau C., Guyomarch J., Robbens J., Paul-Pont I., Soudant P., Huvet A. (2016). Oyster reproduction is affected by exposure to polystyrene microplastics.. Proc. Natl. Acad. Sci. USA.

[234] Qiang L., Cheng J. (2021). Exposure to polystyrene microplastics impairs gonads of zebrafish (Danio rerio).. Chemosphere.

[235] Chatterjee A., Maity S., Banerjee S., Dutta S., Adhikari M., Guchhait R., Biswas C., De S., Pramanick K. (2022). Toxicological impacts of nanopolystyrene on zebrafish oocyte with insight into the mechanism of action: An expression-based analysis.. Sci. Total Environ..

[236] Pitt J.A., Trevisan R., Massarsky A., Kozal J.S., Levin E.D., Di Giulio R.T. (2018). Maternal transfer of nanoplastics to offspring in zebrafish (Danio rerio): A case study with nanopolystyrene.. Sci. Total Environ..

[237] Duan Z., Duan X., Zhao S., Wang X., Wang J., Liu Y., Peng Y., Gong Z., Wang L. (2020). Barrier function of zebrafish embryonic chorions against microplastics and nanoplastics and its impact on embryo development.. J. Hazard. Mater..

[238] Feng M., Luo J., Wan Y., Zhang J., Lu C., Wang M., Dai L., Cao X., Yang X., Wang Y. (2022). Polystyrene nanoplastic exposure induces developmental toxicity by activating the oxidative stress response and base excision repair pathway in zebrafish (Danio rerio).. ACS Omega.

[239] Lin W., Luo H., Wu J., Liu X., Cao B., Liu Y., Yang P., Yang J. (2023). Polystyrene microplastics enhance the microcystin-LR-induced gonadal damage and reproductive endocrine disruption in zebrafish.. Sci. Total Environ..

[240] Tarasco M., Gavaia P.J., Bensimon-Brito A., Cordelières F.P., Santos T., Martins G., de Castro D.T., Silva N., Cabrita E., Bebianno M.J., Stainier D.Y.R., Cancela M.L., Laizé V. (2022). Effects of pristine or contaminated polyethylene microplastics on zebrafish development.. Chemosphere.

[241] Gao Y., Li A., Zhang W., Pang S., Liang Y., Song M. (2022). Assessing the toxicity of bisphenol A and its six alternatives on zebrafish embryo/larvae.. Aquat. Toxicol..

[242] Zhao F., Jiang G., Wei P., Wang H., Ru S. (2018). Bisphenol S exposure impairs glucose homeostasis in male zebrafish (Danio rerio).. Ecotoxicol. Environ. Saf..

[243] Yuan M., Chen S., Zeng C., Fan Y., Ge W., Chen W. (2023). Estrogenic and non-estrogenic effects of bisphenol A and its action mechanism in the zebrafish model: An overview of the past two decades of work.. Environ. Int..

[244] Wang L., Zhu Y., Gu J., Yin X., Guo L., Qian L., Shi L., Guo M., Ji G. (2023). The toxic effect of bisphenol AF and nanoplastic coexposure in parental and offspring generation zebrafish.. Ecotoxicol. Environ. Saf..

[245] Leslie H.A., van Velzen M.J.M., Brandsma S.H., Vethaak A.D., Garcia-Vallejo J.J., Lamoree M.H. (2022). Discovery and quantification of plastic particle pollution in human blood.. Environ. Int..

[246] Wen S., Chen Y., Tang Y., Zhao Y., Liu S., You T., Xu H. (2023). Male reproductive toxicity of polystyrene microplastics: Study on the endoplasmic reticulum stress signaling pathway.. Food Chem. Toxicol..

[247] Zhao T., Shen L., Ye X., Bai G., Liao C., Chen Z., Peng T., Li X., Kang X., An G. (2023). Prenatal and postnatal exposure to polystyrene microplastics induces testis developmental disorder and affects male fertility in mice.. J. Hazard. Mater..

[248] An R., Wang X., Yang L., Zhang J., Wang N., Xu F., Hou Y., Zhang H., Zhang L. (2021). Polystyrene microplastics cause granulosa cells apoptosis and fibrosis in ovary through oxidative stress in rats.. Toxicology.

[249] Deng Y., Yan Z., Shen R., Huang Y., Ren H., Zhang Y. (2021). Enhanced reproductive toxicities induced by phthalates contaminated microplastics in male mice (Mus musculus).. J. Hazard. Mater..

[250] Wei Z., Wang Y., Wang S., Xie J., Han Q., Chen M. (2022). Comparing the effects of polystyrene microplastics exposure on reproduction and fertility in male and female mice.. Toxicology.

[251] Marcelino R.C., Cardoso R.M., Domingues E.L.B.C., Gonçalves R.V., Lima G.D.A., Novaes R.D. (2022). The emerging risk of microplastics and nanoplastics on the microstructure and function of reproductive organs in mammals: A systematic review of preclinical evidence.. Life Sci..

[252] Yuan Y., Qin Y., Wang M., Xu W., Chen Y., Zheng L., Chen W., Luo T. (2022). Microplastics from agricultural plastic mulch films: A mini-review of their impacts on the animal reproductive system.. Ecotoxicol. Environ. Saf..

[253] Maradonna F., Vandenberg L.N., Meccariello R. (2022). Editorial: Endocrine-disrupting compounds in plastics and their effects on reproduction, fertility, and development.. Front. Toxicol..

[254] Wu H., Liu Q., Yang N., Xu S. (2023). Polystyrene-microplastics and DEHP co-exposure induced DNA damage, cell cycle arrest and necroptosis of ovarian granulosa cells in mice by promoting ROS production.. Sci. Total Environ..

[255] Liu Z., Zhuan Q., Zhang L., Meng L., Fu X., Hou Y. (2022). Polystyrene microplastics induced female reproductive toxicity in mice.. J. Hazard. Mater..

[256] Zeng L., Zhou C., Xu W., Huang Y., Wang W., Ma Z., Huang J., Li J., Hu L., Xue Y., Luo T., Zheng L. (2023). The ovarian-related effects of polystyrene nanoplastics on human ovarian granulosa cells and female mice.. Ecotoxicol. Environ. Saf..

[257] Park E.J., Han J.S., Park E.J., Seong E., Lee G.H., Kim D.W., Son H.Y., Han H.Y., Lee B.S. (2020). Repeated-oral dose toxicity of polyethylene microplastics and the possible implications on reproduction and development of the next generation.. Toxicol. Lett..

[258] Wei Y., Zhou Y., Long C., Wu H., Hong Y., Fu Y., Wang J., Wu Y., Shen L., Wei G. (2021). Polystyrene microplastics disrupt the blood-testis barrier integrity through ROS-Mediated imbalance of mTORC1 and mTORC2.. Environ. Pollut..

[259] Jin H., Yan M., Pan C., Liu Z., Sha X., Jiang C., Li L., Pan M., Li D., Han X., Ding J. (2022). Chronic exposure to polystyrene microplastics induced male reproductive toxicity and decreased testosterone levels *via* the LH-mediated LHR/cAMP/PKA/StAR pathway.. Part. Fibre Toxicol..

[260] Hou L., Wang D., Yin K., Zhang Y., Lu H., Guo T., Li J., Zhao H., Xing M. (2022). Polystyrene microplastics induce apoptosis in chicken testis *via* crosstalk between NF-κB and Nrf2 pathways.. Comp. Biochem. Physiol. C Toxicol. Pharmacol..

[261] Hou B., Wang F., Liu T., Wang Z. (2021). Reproductive toxicity of polystyrene microplastics: *In vivo* experimental study on testicular toxicity in mice.. J. Hazard. Mater..

[262] Xie X., Deng T., Duan J., Xie J., Yuan J., Chen M. (2020). Exposure to polystyrene microplastics causes reproductive toxicity through oxidative stress and activation of the p38 MAPK signaling pathway.. Ecotoxicol. Environ. Saf..

[263] Zhou Y., Xu W., Yuan Y., Luo T. (2020). What is the Impact of Bisphenol A on sperm function and related signaling pathways: A Mini-review?. Curr. Pharm. Des..

[264] Sui A., Yao C., Chen Y., Li Y., Yu S., Qu J., Wei H., Tang J., Chen G. (2023). Polystyrene nanoplastics inhibit StAR expression by activating HIF-1α *via* ERK1/2 MAPK and AKT pathways in TM3 Leydig cells and testicular tissues of mice.. Food Chem. Toxicol..

[265] Jin H., Ma T., Sha X., Liu Z., Zhou Y., Meng X., Chen Y., Han X., Ding J. (2021). Polystyrene microplastics induced male reproductive toxicity in mice.. J. Hazard. Mater..

[266] Sun Z., Wen Y., Zhang F., Fu Z., Yuan Y., Kuang H., Kuang X., Huang J., Zheng L., Zhang D. (2023). Exposure to nanoplastics induces mitochondrial impairment and cytomembrane destruction in leydig cells.. Ecotoxicol. Environ. Saf..

[267] Mruk D.D., Cheng C.Y. (2015). The mammalian blood-testis barrier: Its biology and regulation.. Endocr. Rev..

[268] Xu W., Yuan Y., Tian Y., Cheng C., Chen Y., Zeng L., Yuan Y., Li D., Zheng L., Luo T. (2023). Oral exposure to polystyrene nanoplastics reduced male fertility and even caused male infertility by inducing testicular and sperm toxicities in mice.. J. Hazard. Mater..

[269] Hu R., Yao C., Li Y., Qu J., Yu S., Han Y., Chen G., Tang J., Wei H. (2022). Polystyrene nanoplastics promote CHIP-mediated degradation of tight junction proteins by activating IRE1α/XBP1s pathway in mouse Sertoli cells.. Ecotoxicol. Environ. Saf..

[270] Hassine M.B.H., Venditti M., Rhouma M.B., Minucci S., Messaoudi I. (2023). Combined effect of polystyrene microplastics and cadmium on rat blood-testis barrier integrity and sperm quality.. Environ. Sci. Pollut. Res. Int..

[271] Venditti M., Ben Hadj Hassine M., Messaoudi I., Minucci S. (2023). The simultaneous administration of microplastics and cadmium alters rat testicular activity and changes the expression of PTMA, DAAM1 and PREP.. Front. Cell Dev. Biol..

[272] Liu J., Ma M., Zhu D., Xia T., Qi Y., Yao Y., Guo X., Ji R., Chen W. (2018). Polystyrene nanoplastics-enhanced contaminant transport: Role of irreversible adsorption in glassy polymeric domain.. Environ. Sci. Technol..

[273] Li D., Sun W., Jiang X., Yu Z., Xia Y., Cheng S., Mao L., Luo S., Tang S., Xu S., Zou Z., Chen C., Qiu J., Zhou L. (2022). Polystyrene nanoparticles enhance the adverse effects of di-(2-ethylhexyl) phthalate on male reproductive system in mice.. Ecotoxicol. Environ. Saf..

[274] Cui H., Yang W., Cui Y., Qi L., Jiang X., Li M. (2023). Adverse effects of pristine and aged polystyrene microplastics in mice and their Nrf2-mediated defense mechanisms with tissue specificity.. Environ. Sci. Pollut. Res. Int..

[275] Liu T., Hou B., Zhang Y., Wang Z. (2022). Determination of biological and molecular attributes related to polystyrene microplastic-induced reproductive toxicity and its reversibility in male mice.. Int. J. Environ. Res. Public Health.

[276] Rizwan A., Ijaz M.U., Hamza A., Anwar H. (2023). Attenuative effect of astilbin on polystyrene microplastics induced testicular damage: Biochemical, spermatological and histopathological-based evidences.. Toxicol. Appl. Pharmacol..

[277] Ijaz M.U., Najam S., Hamza A., Azmat R., Ashraf A., Unuofin J.O., Lebelo S.L., Simal-Gandara J. (2023). Pinostrobin alleviates testicular and spermatological damage induced by polystyrene microplastics in adult albino rats.. Biomed. Pharmacother..

[278] Hamza A., Ijaz M.U., Anwar H. (2023). Rhamnetin alleviates polystyrene microplastics-induced testicular damage by restoring biochemical, steroidogenic, hormonal, apoptotic, inflammatory, spermatogenic and histological profile in male albino rats.. Hum. Exp. Toxicol..

[279] D’Angelo S., Scafuro M., Meccariello R. (2019). BPA and nutraceuticals, simultaneous effects on endocrine functions.. Endocr. Metab. Immune Disord. Drug Targets.

[280] Kim S., Kim H., Yim Y.S., Ha S., Atarashi K., Tan T.G., Longman R.S., Honda K., Littman D.R., Choi G.B., Huh J.R. (2017). Maternal gut bacteria promote neurodevelopmental abnormalities in mouse offspring.. Nature.

[281] Han V.X., Patel S., Jones H.F., Dale R.C. (2021). Maternal immune activation and neuroinflammation in human neurodevelopmental disorders.. Nat. Rev. Neurol..

[282] Schwabl P., Köppel S., Königshofer P., Bucsics T., Trauner M., Reiberger T., Liebmann B. (2019). Detection of various microplastics in human stool.. Ann. Intern. Med..

[283] Xu J.L., Lin X., Wang J.J., Gowen A.A. (2022). A review of potential human health impacts of micro- and nanoplastics exposure.. Sci. Total Environ..

[284] Wu P., Lin S., Cao G., Wu J., Jin H., Wang C., Wong M.H., Yang Z., Cai Z. (2022). Absorption, distribution, metabolism, excretion and toxicity of microplastics in the human body and health implications.. J. Hazard. Mater..

[285] La Merrill M.A., Vandenberg L.N., Smith M.T., Goodson W., Browne P., Patisaul H.B., Guyton K.Z., Kortenkamp A., Cogliano V.J., Woodruff T.J., Rieswijk L., Sone H., Korach K.S., Gore A.C., Zeise L., Zoeller R.T. (2020). Consensus on the key characteristics of endocrine-disrupting chemicals as a basis for hazard identification.. Nat. Rev. Endocrinol..

